# Interstitial and recruited macrophages prevent tuberculosis relapse by limiting immune evasion

**DOI:** 10.1038/s44321-026-00432-6

**Published:** 2026-04-29

**Authors:** Valerie Vinette, Anthony Castro, Heather Kim, Carolina Trujillo, Min Xie, Martin Gengenbacher, Thomas R Ioerger, Sabine Ehrt

**Affiliations:** 1 Department of Microbiology and Immunology, Weill Cornell Medicine, New York, NY USA; 2 Center for Discovery and Innovation, Hackensack Meridian Health, Nutley, NJ USA; 3 Hackensack Meridian School of Medicine, Nutley, NJ USA; 4 Department of Computer Science and Engineering, Texas A&M University, College Station, TX USA

**Keywords:** Immunology, Microbiology, Virology & Host Pathogen Interaction

## Abstract

Alveolar macrophages are the first immune cells to encounter *Mycobacterium tuberculosis* (Mtb) in the lungs, but they frequently fail to eliminate this pathogen, allowing Mtb to persist and replicate. Interstitial macrophages (IMs) are enlisted to restrict bacterial growth and limit immune evasion. While IMs have been implicated in controlling acute Mtb infection, their role during latent tuberculosis infection (LTBI) remains unexplored. To address this, we utilized a previously established mouse model of paucibacillary Mtb infection that recapitulates key aspects of human LTBI to deplete IMs during the latent phase. Depletion of IMs and recruited macrophages (RMs) led to TB relapse in 26% of mice compared to 2% in controls. Mice that relapsed exhibited an increased proportion of pro-inflammatory IMs and elevated concentrations of G-CSF, GM-CSF, IL-3, IL-12, IL-13, IL-17A, and KC in the lung. These findings demonstrate that IMs and RMs play a critical role in controlling latent Mtb and preventing TB relapse.

## Synopsis

The transient depletion of interstitial and recruited macrophages in a mouse model of tuberculosis latency results in increased tuberculosis relapse and is accompanied by elevated pro-inflammatory interstitial macrophages and cytokines in the lung.

- Paucibacillary Mtb infection was accompanied by sustained IL-6, IL-10 and TNF concentrations in lungs.
- Depletion of interstitial and recruited macrophages was accompanied by increased neutrophil recruitment to the lung and resulted in increased TB relapse.
- TB relapse was accompanied by elevated pro-inflammatory interstitial macrophages and cytokines in the lung.

## Abstract (web summary)

The transient depletion of interstitial and recruited macrophages in a mouse model of tuberculosis latency results in increased tuberculosis relapse and is accompanied by elevated pro-inflammatory interstitial macrophages and cytokines in the lung.

## Graphical abstract

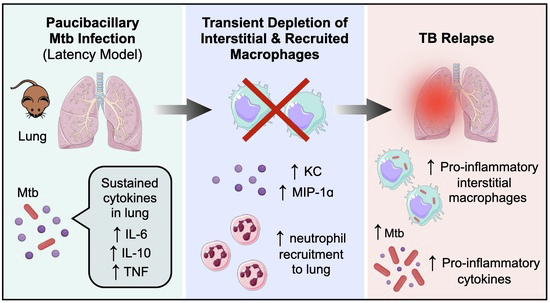

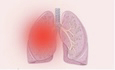

The paper explained **Problem**

Tuberculosis (TB) remains the world’s deadliest infectious disease, killing 1.3 million people in 2023 alone. One of the most challenging aspects of TB is that the majority of infected individuals do not develop active disease but instead harbor *Mycobacterium tuberculosis* (Mtb) in a dormant, symptom-free state known as latent TB infection. Under certain conditions, such as immune suppression, this latent infection can reactivate and cause life-threatening disease. A central but unresolved question is which immune cells are responsible for keeping the bacteria controlled during latency?

**Results**

Using a mouse model of latent TB infection, we demonstrate that transient depletion of interstitial and recruited lung macrophages reactivates Mtb and promotes tuberculosis relapse. During latency, a low-grade but persistent inflammatory state characterized by sustained IL-6, TNF, and IL-10 appears to contribute to Mtb control, while TB relapse is accompanied by elevated pro-inflammatory interstitial macrophages and cytokines.

**Impact**

Our findings establish IMs and RMs as indispensable for Mtb control during latent tuberculosis infection. Strategies to boost or preserve these cell populations in at-risk individuals (e.g., those living with HIV or receiving immunosuppression) could be an approach to preventing Mtb reactivation.

## Introduction

Tuberculosis (TB) is the deadliest infectious disease in the world, affecting 10.8 million people annually and causing 1.3 million deaths in 2023 (WHO, 2024). Approximately 95% of people exposed to *Mycobacterium tuberculosis* (Mtb) do not develop the disease. Some individuals develop latent TB infection (LTBI), in which the bacteria persist without causing any clinical symptoms. Sometimes, the pathogen reactivates and causes active TB. Following inhalation, Mtb is first encountered by tissue resident alveolar macrophages (AMs) in the lung. While AMs are the initial line of defense, they frequently fail to eliminate intracellular Mtb, enabling bacterial persistence and potential dissemination. Interstitial macrophages (IMs), located within the lung interstitium, are recruited to the site of infection to control Mtb and restrict their evasion from the immune system. IMs are more pro-inflammatory in nature (Pisu et al, 2020; Pisu et al, 2021) and more restrictive for Mtb than AMs (Huang et al, 2018). In a mouse model of acute TB, IM depletion during the early stage of infection promoted the growth of Mtb in the lung, highlighting their importance in the early immune response to Mtb infection (Huang et al, 2018). The cellular control of LTBI is multifactorial and depends on a delicate immunological balance that prevents Mtb reactivation without causing excessive inflammation. Central to this process is the formation and maintenance of granulomas, organized immune structures composed primarily of macrophages, but also containing T cells and other immune cells (Dutta et al, 2014; Flynn et al, 2011; McCaffrey et al, 2022; Silva Miranda et al, 2012). Within granulomas, macrophages provide the cellular niche where Mtb can persist in a non-replicating or slowly replicating state (Flynn et al, 2011; McCaffrey et al, 2022). T cell-mediated control by both CD4^+^ and CD8^+^ cells is also important in controlling Mtb, in part by activating infected macrophages (Adekambi et al, 2015; Hou et al, 2021). However, the specific role of IMs in controlling latent Mtb infection remains unexplored. To assess the importance of IMs for Mtb control during latency, we used a paucibacillary mouse model that recapitulates key aspects of human LTBI and yields reproducible relapse frequencies across independent experiments (Su et al, 2021). We previously demonstrated that transient genetic depletion of the essential Mtb protein biotin protein ligase (BPL) leads to apparent sterilization of Mtb and establishes paucibacillary Mtb infection in mice, followed by spontaneous reactivation in about 30% of mice 10 months post-infection (Su et al, 2021). Given that macrophages serve as the primary reservoir for Mtb and that IMs are important in restricting Mtb during acute infection, we hypothesized that depleting IMs during the paucibacillary, latent phase would result in increased bacterial growth and elevated TB relapse rates.

Here, we demonstrate that transient depletion of IMs together with RMs during latency promotes TB relapse in mice. Longitudinal cytokine expression analyses and flow cytometry immunoprofiling performed in parallel revealed an increased frequency of pro-inflammatory IMs and an altered cytokine milieu in mice with TB relapse. These data indicate that IMs and RMs are critical for restraining persistent Mtb, maintaining latency, and limiting TB reactivation and disease.

## Results and discussion

### The pulmonary myeloid cell compartment and cytokine milieu are altered in a paucibacillary mouse model of latent tuberculosis infection

**Figure 1:**
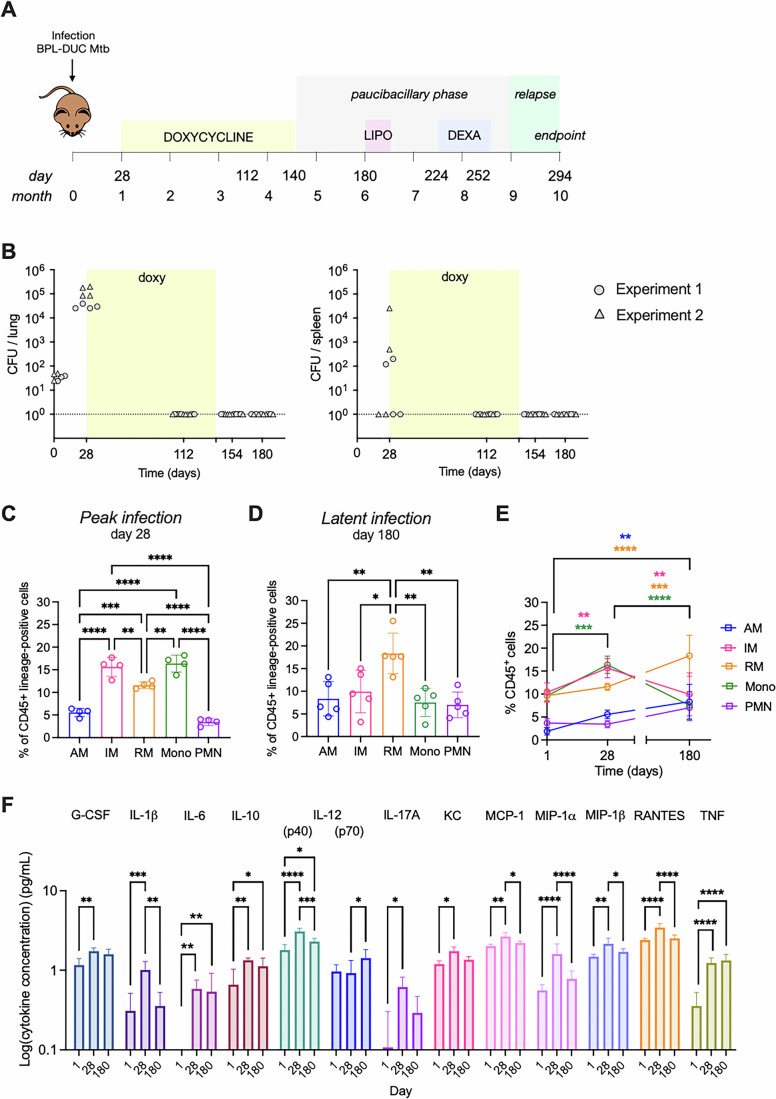
The pulmonary myeloid cell compartment and cytokine milieu are altered in a paucibacillary mouse model of latent tuberculosis infection. (**A**) Study design. Mice were infected with Mtb BPL-DUC by aerosol. Doxycycline (yellow) was administered for 16 weeks to deplete BPL, kill the bacteria, and attain the paucibacillary phase (gray). Clodronate liposomes or control liposomes (lipo) were injected intravenously every two or three days for 2 weeks to deplete interstitial macrophages (IMs) (pink). One month after treatment, dexamethasone (dexa) was administered to a group of mice treated with control liposomes for 1 month (blue). Relapse was assessed at 3 months post-depletion on days 287-302, with 294 depicted as mean. (**B**) Bacterial burden was assessed throughout the infection by colony-forming units (CFU) determinations in the lung and spleen of mice. Different symbols (circle, triangle) denote data from two independent experiments that were combined for analysis. (**C**, **D**) Immunoprofiling was performed by flow cytometry with lung homogenates to assess the frequency of alveolar macrophages (AM), interstitial macrophages (IM) recruited macrophages (RM), monocytes (Mono), and neutrophils (PMN) at the peak of infection (**C**) and during the paucibacillary phase, prior to treatment with liposomes (**D**). (**E**) Frequency of macrophage, monocyte, and neutrophil populations in the lung over time before treatment with liposomes. (**F**) Cytokine concentrations in the lung of mice at days 1, 28, and 180. Data represent the mean ± SD of four to five biological replicates. *P < 0.05, **P < 0.01, ***P < 0.001, ****P < 0.0001 based on one-way ANOVA (**B**, **C**) or two-way ANOVA (**D**–**F**) statistical test with Tukey’s multiple comparisons tests. Exact *n* and *P* values are described in Appendix Fig. S1. Source data are available online for this figure.

Considering the enhanced ability of IMs to restrict Mtb during acute infection, we sought to study their importance in controlling persisting Mtb during latency in mice. We adapted our previously described paucibacillary mouse model of LTBI (Su et al, 2021) by adding a 2-week treatment with clodronate-loaded or control liposomes during the latent phase to assess the importance of IMs in preventing TB relapse (Fig. 1A). As in our previous studies, Mtb BPL-DUC replicated during the first month of infection and was undetectable in lungs and spleens following a 16-week doxycycline (doxy) treatment due to the depletion of BPL causing death of Mtb (Fig. 1B).

**Figure EV1:**
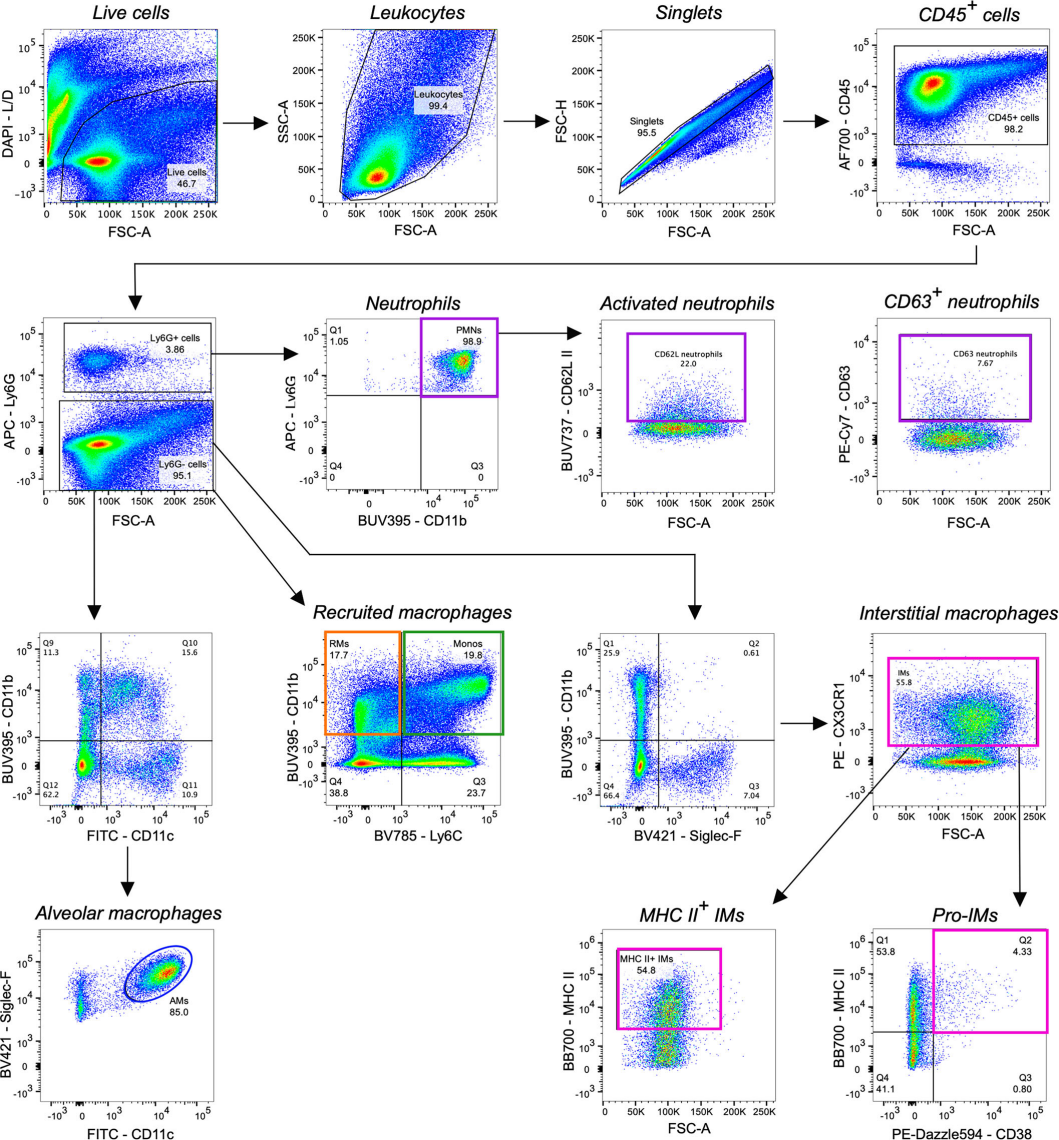
Gating strategy to identify myeloid cell populations. Gating strategy to identify neutrophils (PMNs, purple), alveolar macrophages (AMs, blue), recruited macrophages (RMs, orange), monocytes (Monos, green), and interstitial macrophages (IMs, pink) by flow cytometry.

We characterized the pulmonary immune cell landscape by flow cytometry throughout the course of the experiment and identified alveolar macrophages (AMs: Ly6G^−^ CD11b^−^ CD11c^+^ Siglec-F^+^), interstitial macrophages (IMs: Ly6G^−^ CD11b^+^ Siglec-F^−^ CX3CR1^+^), recruited macrophages (RMs: Ly6G^−^ CD11b^+^ Ly6C^−^), monocytes (Monos: Ly6G^−^ CD11b^+^ Ly6C^+^) and neutrophils (PMNs: Ly6G^+^ CD11b^+^) from CD45^+^ cells, as described in our gating strategy (Fig. EV1). At the peak of infection on day 28, we found that the largest proportions of cells in the lung were IMs and monocytes (Fig. 1C), while RMs represented the predominant immune cell type during the latent phase of infection on day 180 (Fig. 1D). Analyzing the changes of cell population frequencies over time revealed that AMs and neutrophils remained at relatively low levels throughout the infection, while IMs and monocytes increased during the first 4 weeks of infection and decreased to baseline levels alongside the elimination of Mtb. In contrast, RMs were continuously recruited, likely to support and replenish pre-existing IMs, and represented the most abundant immune cell type during latency (Fig. 1E).

To assess the activity of pulmonary immune cells, we measured the concentrations of various cytokines in the lungs of mice at days 1, 28, and 180 post-infection. The levels of several pro-inflammatory cytokines were increased at the peak of infection at day 28 compared to uninfected mice (Fig. 1F). Among these, IL-6, IL-10, and TNF remained elevated during latency as determined on day 180. In contrast, IL-1β, MIP-1ɑ, MIP-1β, and RANTES returned to baseline levels during the latent phase. IL-12p70 was the only cytokine that increased in the lung during latency compared to peak infection (Fig. 1F). The sustained IL-6, IL-10, and TNF concentrations throughout latency, with IL-6 and TNF showing the largest increase from before infection (Fig. 1F), suggest that this low-grade inflammatory state is essential for Mtb control while preventing excessive immune cell recruitment.

### Clodronate liposome treatment during latency leads to a depletion of interstitial and recruited macrophages, and increased recruitment of neutrophils to the lung

**Figure 2:**
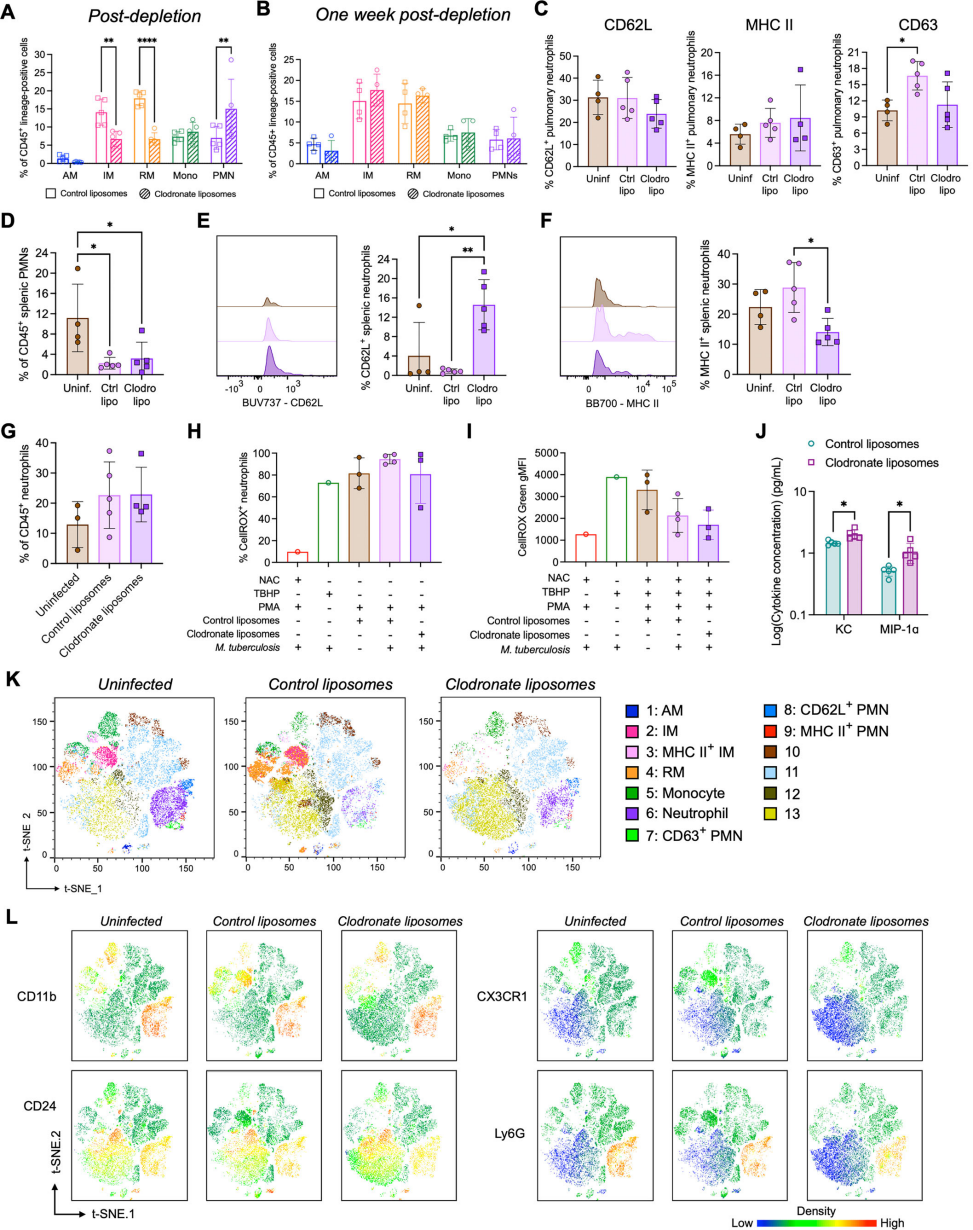
Clodronate liposome treatment during LTBI leads to a depletion of interstitial and recruited macrophages and increased recruitment of neutrophils to the lung. Mice were treated with control or clodronate liposomes for 2 weeks during the paucibacillary phase, and lung (**A**–**C**), spleen (**D**–**F**), and blood (**G**–**I**) were analyzed. (**A**, **B**) Immunoprofiling was performed by flow cytometry with lung homogenates to assess the frequency of alveolar macrophages (AM), interstitial macrophages (IM), recruited macrophages (RM), monocytes (Mono), and neutrophils (PMN) immediately post-depletion with liposomes (**A**) and one week post-depletion (**B**). (**C**) Flow cytometry from lung homogenates following liposome treatment was done to assess the frequency of neutrophils expressing the activation markers CD62L and MHC II, and the degranulation marker CD63 in uninfected mice (Uninf) and infected mice treated with control liposomes (Ctrl lipo) or clodronate liposomes (Clodro lipo). (**D**–**F**) Flow cytometry from spleen homogenates following liposome treatment was performed to assess the frequency of neutrophils (**D**) and the fraction of these neutrophils expressing the activation markers CD62L (**E**) or MHC II (**F**). (**G**) Blood was collected from mice after IM/RM depletion to assess the frequency of circulating neutrophils. (**H**, **I**) ROS assay performed with blood to assess the frequency of ROS-producing neutrophils (**H**) and the geometric mean fluorescence intensity (gMFI) quantifying ROS production from neutrophils (**I**). (**J**) Quantification of cytokine concentrations in the lung of mice treated with control or clodronate liposomes immediately following liposome treatment. (**K**) t-SNE plots of cell subtypes after clustering, colored according to cellular identity and separated by condition immediately following treatment in uninfected mice, and infected mice treated with control or clodronate liposomes. (**L**) Multigraph color mapping was performed with flow cytometry data from uninfected mice and infected mice treated with control or clodronate liposomes to visualize the expression of CD11b, CD24, CX3CR1, and Ly6G across clusters. Data represent the mean ± SD of three to five biological replicates. *P < 0.05, **P < 0.01, ****P < 0.0001 based on one-way ANOVA (for **C**–**I**) or two-way ANOVA statistical test with Tukey’s (for **A**, **B**, **K**) or Šídák’s (**J**) multiple comparisons test. Exact *n* and *P* values are described in Appendix Fig. S2. Source data are available online for this figure.

Having established a paucibacillary, latent phase of TB infection, we treated infected mice with control or clodronate liposomes for 2 weeks to deplete IMs (Fig. 1A). We performed immunoprofiling of the myeloid cell compartment in the lung (Fig. 2A,C), spleen (Fig. 2D–F), and blood (Fig. 2G–I) immediately post-depletion. As expected, clodronate treatment significantly reduced the frequency of IMs in the lung compared to control liposome treatment (Fig. 2A). Not surprisingly, the frequency of RMs was similarly reduced in the lung of clodronate liposome-treated mice, as liposomes injected intravenously are distributed through the bloodstream, where they encounter RMs before they differentiate into IMs in the lung (Fig. 2A). In contrast, AMs were not significantly affected by the intravenous administration of clodronate liposomes. Surprisingly, IM depletion resulted in a twofold increase in the number of neutrophils recruited to the lung (Fig. 2A). One week after depletion, all monocyte, macrophage, and neutrophil populations had returned to homeostasis, as indicated by the similar cell population frequencies between mice treated with control or clodronate liposomes (Fig. 2B).

A recent study demonstrated that clodronate liposomes can induce neutrophil “stunning”, a transient state of functional suppression, blocking phagocytosis, cytokine expression, ROS production, and migration, observed in a serum transfer arthritis model (Culemann et al, 2023; Leliefeld et al, 2016). We therefore assessed neutrophil phenotype and function in our model. The transiently recruited neutrophils displayed no differences in expression of the activation markers CD62L and MHC II or the degranulation marker CD63, regardless of liposome treatment (Fig. 2C), indicating functional similarity to controls. We also assessed neutrophil abundance, activation, and functionality in the spleen and blood. Immunoprofiling revealed that although the number of neutrophils in spleens of infected mice was reduced during latency compared to uninfected mice, clodronate liposome treatment had no effect on neutrophil numbers (Fig. 2D). However, mice treated with clodronate liposomes exhibited an increased frequency of CD62L^+^ neutrophils (Fig. 2E), suggesting either reduced activation or a transitional phenotype (Drescher et al, 2020; Leliefeld et al, 2016; Malengier-Devlies et al, 2021; Paudel et al, 2022). They further exhibited a decreased frequency of MHC II^+^ splenic neutrophils (Fig. 2F), indicating reduced maturation (Borkute et al, 2021; Forrer et al, 2024; Vono et al, 2017). IM and RM depletion may lead to systemic immune dysregulation, triggering emergency granulopoiesis or systemic inflammation (Bain and MacDonald, 2022; Bedoret et al, 2009; Hou et al, 2021; Tamoutounour et al, 2013; Zaynagetdinov et al, 2013). It is possible that IMs indirectly regulate neutrophil maturation and that their depletion could disrupt immunoregulatory cues (Dang et al, 2023; Maier-Begandt et al, 2024; Su et al, 2020).

In the circulation, we found no difference in the proportion of neutrophils between uninfected mice and infected mice treated with either liposome, as assessed by immunoprofiling blood (Fig. 2G). To investigate the functionality of these neutrophils, we quantified reactive oxygen species (ROS) production, which revealed no difference in the frequency of ROS-producing neutrophils (Fig. 2H) or in the amount of ROS produced by neutrophils (Fig. 2I), whether they were treated with control or clodronate liposomes. These data indicate that neutrophils isolated from clodronate liposome-treated mice were as functionally active as those from control mice.

**Figure EV2:**
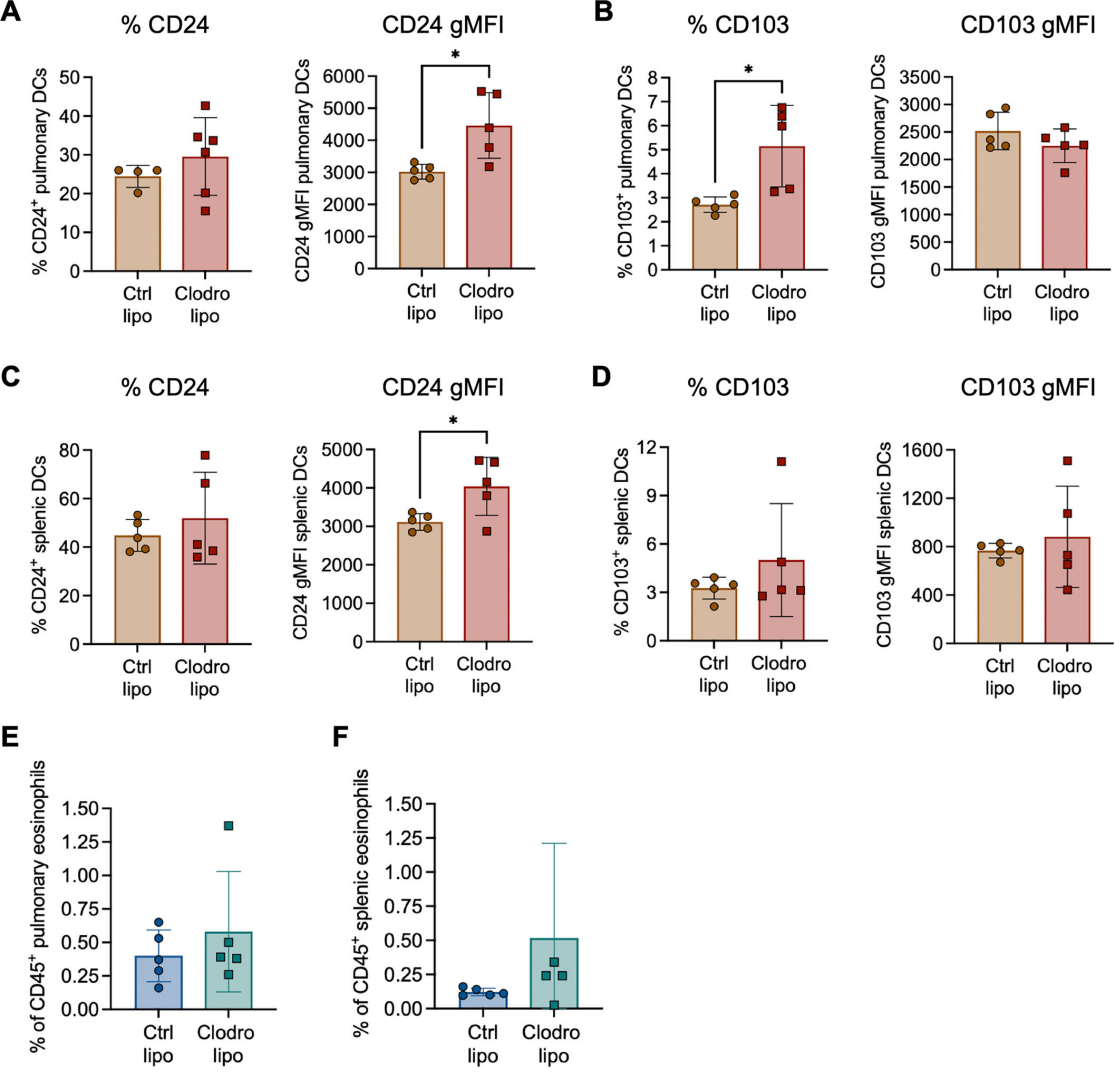
Immunophenotype of dendritic cells and eosinophils in the lung and spleen upon interstitial and recruited macrophage depletion during LTBI. Lung and spleen were harvested from mice treated with control or clodronate liposomes immediately following depletion of interstitial macrophages. (**A**–**D**) Immunoprofiling was performed by flow cytometry to assess pulmonary CD11c^+^ DCs and their frequency and gMFI of CD24 (**A**) and CD103 (**B**), as well as the frequency of splenic CD11c^+^ DCs for CD24 (**C**) and CD103 (**D**). (**E**, **F**) Immunoprofiling was performed by flow cytometry to assess the frequency of pulmonary and splenic eosinophils, identified as CD11b^+^ Siglec-F^+^ cells. Data represent mean ± SD of five biological replicates. *P < 0.05 based on an unpaired *t* test. Exact *P* values are described in Appendix Fig. S5. Source data are available online for this figure.

Considering the proposed roles of dendritic cells (Abrahem et al, 2020; Kim et al, 2025) and eosinophils (Bohrer et al, 2021; Bohrer et al, 2022) in Mtb control, we characterized these populations in the lung and spleen immediately following IM depletion. The frequency of CD24^+^CD11c^+^ pulmonary and splenic dendritic cells (DCs) remained unchanged; however, CD11c^+^ DCs exhibited increased CD24 expression when IMs and RMs were depleted (Fig. EV2A,C). Clodronate liposome-treated mice also exhibited an increased frequency of CD103^+^ CD11c^+^ DCs in the lung (Fig. EV2B), but not in the spleen (Fig. EV2D). These data suggest a potential shift toward cross-presenting, migratory conventional type 1 DCs (cDC1) in the lung after IM/RM depletion, which could enhance bacterial control or limit excessive inflammation. Additional studies are needed to validate these findings. The frequency of CD11b^+^ Siglec-F^+^ eosinophils remained unchanged in both the lung (Fig. EV2E) and spleen (Fig. EV2F) following clodronate liposome treatment.

### Cytokine microenvironment and immune landscape remodeling during LTBI

**Figure EV3:**
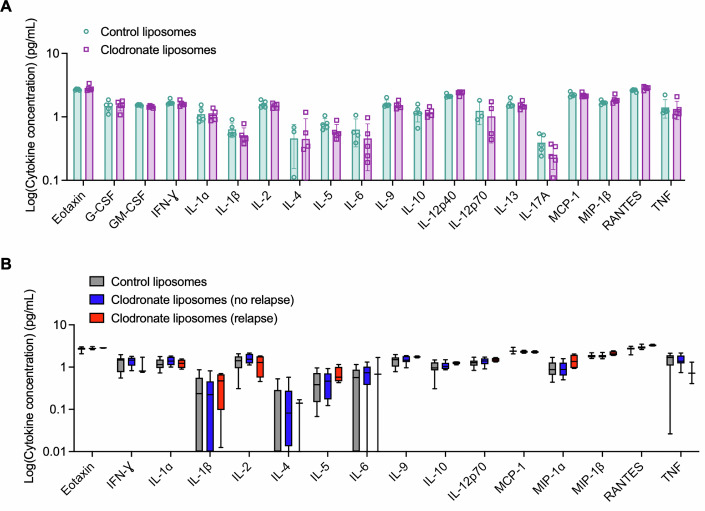
Pulmonary cytokine levels after treatment with liposomes. Quantification of cytokine concentrations in the lung of mice treated with control or clodronate liposomes immediately following treatment (**A**) and at experimental endpoint (**B**). For (**A**), data represent the mean ± SD of five biological replicates. Statistical analysis performed by a two-way ANOVA statistical test with Tukey’s multiple comparisons test. For (**B**), data represent the mean ± SD of 22 (control liposomes), 14 (clodronate liposomes, no relapse) or 4 (clodronate liposome, relapse) biological replicates. Statistical analysis performed by *F* test. Box plots depict the interquartile range (IQR), extending from the 25th to the 75th percentiles of the data, and the central line in the middle of each box indicates the median value. Whiskers extend the full range of the data (min to max). Source data are available online for this figure.

We next assessed the cytokine microenvironment by measuring cytokine levels in the lungs. Of all the cytokines analyzed, only KC and MIP-1ɑ were elevated in the lungs of mice treated with clodronate liposomes (Fig. 2J), while all other cytokines remained at comparable levels in mice treated with control or clodronate liposomes (Fig. EV3A).

To comprehensively visualize immune landscape remodeling during latency following IM/RM depletion, we performed high-dimensional reduction and clustering analysis. Using our immune cell flow cytometry panel, we identified 12 main cell clusters, as demonstrated by the t-SNE plots (Fig. 2K). In infected mice that were treated with control liposomes, IM (cluster 2) and RM (cluster 4) populations were enriched compared to uninfected mice; these populations were nearly absent in mice treated with clodronate liposomes. Conversely, the neutrophil cluster (cluster 6) expanded upon clodronate treatment (Fig. 2K). This high-dimensional reduction analysis highlights the immune landscape reprogramming that occurs upon IM and RM depletion. Additional markers are required to make a strong call on clusters 10-13, but based on the markers that were excluded from these clusters, they likely represent T cells (cluster 10), MHC II^mid^ B cells (cluster 12) and MHC II^hi^ B cells (cluster 13), with cluster 11 representing an undefined cluster (Fig. 2K). Visualization of individual flow cytometry markers via multigraph color mapping of the t-SNE plots revealed a marked reduction in CD11b and CX3CR1 expression within the IM cluster (cluster 2) upon clodronate liposome treatment (Fig. 2L).

While most cytokines remained comparable between IM/RM-depleted and control mice, KC and MIP-1α levels were elevated approximately fivefold in IM/RM-depleted lungs (Fig. 2K), providing mechanistic insight into the observed neutrophil recruitment. Both chemokines are potent neutrophil chemoattractants (De Filippo et al, 2008; Hilda et al, 2014; Johnson et al, 2011; Ritzman et al, 2010; Tumpey et al, 2002), while MIP-1α additionally promotes recruitment of monocytes, T cells, and dendritic cells via secretion from activated macrophages (Bhavsar et al, 2015; Madsen et al, 2003). Beyond chemotaxis, MIP-1α enhances macrophage phagocytosis and microbial killing and coordinates lesion formation to contain Mtb (Saukkonen et al, 2002; Silva Miranda et al, 2012). The selective elevation of these chemokines likely represents a compensatory mechanism by residual myeloid cells to counterbalance the absence of IMs and RMs, demonstrating the lung’s capacity for rapid immune adaptation when critical cell populations are depleted.

### Depletion of interstitial and recruited macrophages during latency leads to increased tuberculosis relapse, accompanied by elevated pro-inflammatory interstitial macrophages and cytokines in the lung

**Figure 3:**
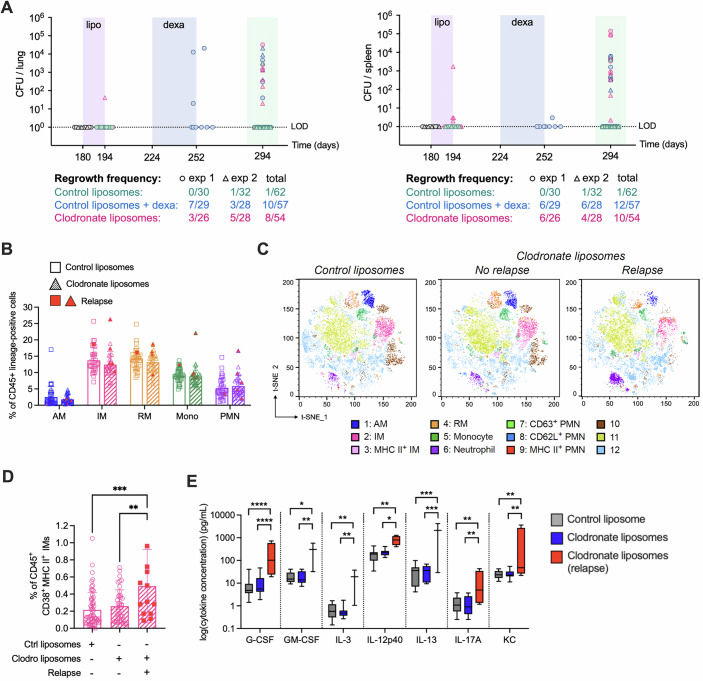
Depletion of interstitial and recruited macrophages during latency results in increased tuberculosis relapse in mice, accompanied by elevated pro-inflammatory interstitial macrophages and cytokines in the lung. (**A**) Bacterial burden before and after IM depletion was assessed by CFU determinations in the lung and spleen of mice. Different symbols (circle, triangle) denote data from two independent experiments that were combined for analysis. Numbers below each graph indicate the frequency of Mtb regrowth in mice treated with control liposomes with or without dexa, or with clodronate liposomes. (**B**) Immunoprofiling performed by flow cytometry 3 months post-depletion to assess the frequency of each immune cell type in the lung of mice treated with control and clodronate liposomes, where the red symbols indicate mice that relapsed. (**C**) t-SNE plots of cell subtypes after clustering, colored according to cellular identity and separated by condition immediately following treatment, namely control liposomes, and mice treated with clodronate liposomes with or without relapse. (**D**) Frequency of pro-inflammatory IMs (CD38^+^ MHC II^+^ IMs) present in the lung of treated mice 3 months post-depletion as assessed by flow cytometry. (**E**) Cytokine concentrations in lung homogenates from mice treated with control liposomes (gray), or from clodronate liposome-treated mice that relapsed (red) or did not relapse (blue). Box plots depict the interquartile range (IQR), extending from the 25th to the 75th percentiles of the data, and the central line in the middle of each box indicates the median value. Whiskers extend the full range of the data (min to max). Data represent the mean ± SD of 4 to 32 biological replicates. *P < 0.05, **P < 0.01, ***P < 0.001, ****P < 0.0001 based on Poisson test (**A**), two-way ANOVA (**B**), one-way ANOVA (**D**) statistical test with Tukey’s multiple comparisons test or *F* test (for **E**). Exact *n* and *P* values are described in Appendix Fig. S3. Source data are available online for this figure.

Throughout the chronic infection, we assessed bacterial burden in the lungs and spleens of mice. As expected, mice immunosuppressed with dexamethasone exhibited bacterial regrowth in both organs, corresponding to a 28% reactivation frequency compared to 2% in mice treated with control liposomes (Fig. 3A; Table 1). Notably, in mice depleted of IMs and RMs, Mtb also reactivated in the lung and spleen, corresponding to a frequency of 26% TB relapse (Table 1). These data demonstrate that transient depletion of IMs and RMs during the paucibacillary phase phenocopies the effect of dexamethasone-induced immunosuppression, leading to enhanced TB relapse. We cannot, however, exclude the possibility that transient neutrophil expansion contributed to relapse, as neutrophils can serve as permissive cellular niches for Mtb (Lovewell et al, 2021; Eum et al, 2010). Future studies could investigate the impact of neutrophil depletion during latency to test their role in preventing Mtb reactivation.

**Table 1:** Analysis of Mtb regrowth and relapse frequencies.

	Control liposomes	Dexamethasone-induced	Clodronate liposomes
Experiments	2	2	2
Mice	62	57	54
Mice with regrowth in the lung (CFU ≥ 1)	1	10	8
Mice with regrowth in the spleen (CFU ≥ 1)	1	12	10
Mice relapsed, CFU in lung or spleen	1	16	14
Relapse frequency (%)	1.6	28.1	25.9
*P* value, compared to control	n/a	2.13E-07	2.73E-04
*P* value, compared to dexamethasone	2.13E-07	n/a	>0.05

Data from two independent experiments, including the number of mice, frequency of regrowth in the lung and in the spleen as assessed by colony-forming units (CFUs), number of mice that relapsed, frequency of relapse, and statistics. The Poisson model was used to evaluate statistical differences in reactivation rates.

**Figure EV4:**
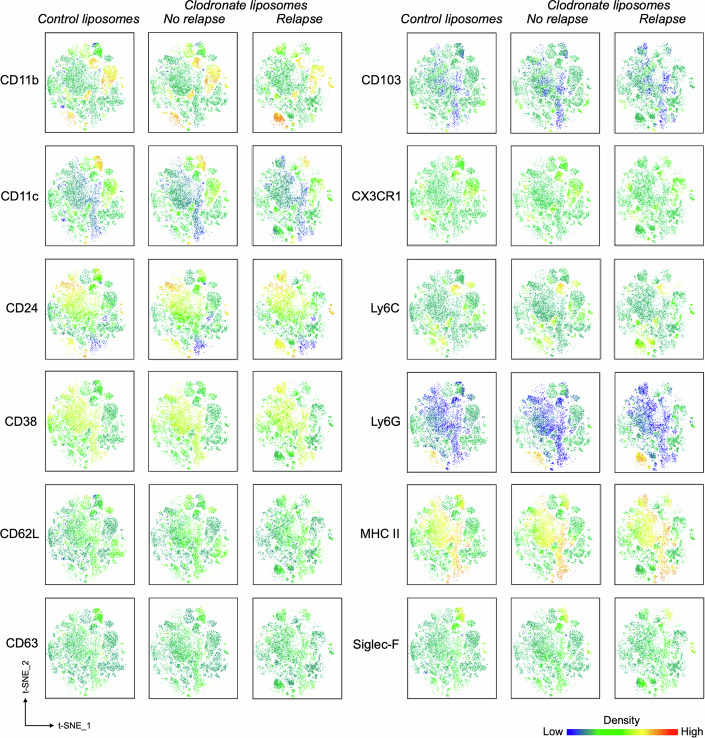
Cell surface markers are expressed at similar levels at the experimental endpoint. Multigraph color mapping was performed with flow cytometry data from uninfected mice and infected mice treated with control or clodronate liposomes to visualize the expression of all flow cytometry markers tested across the various t-SNE clusters.

To characterize the dynamic nature of the myeloid compartment, we performed immunoprofiling of pulmonary myeloid cells by flow cytometry at the study endpoint (10 months post-infection). Three months after liposome depletion, all myeloid populations assessed — AMs, IMs, RMs, monocytes, and neutrophils—had returned to baseline pre-depletion levels (Fig. 3B). Comparison of t-SNE plots between control and clodronate-treated mice that relapsed or did not relapse revealed no major changes in cell cluster composition (Fig. 3C), emphasizing the return to homeostasis of the analyzed immune cell populations. Clusters 10-12 remain incompletely defined, but likely represent T cells (cluster 10) and B cells (cluster 11), with cluster 12 being undefined. Multigraph color mapping of individual flow cytometry markers on t-SNE plots confirmed that most markers and cell populations returned to similar expression levels across the three experimental groups (Fig. EV4).

Despite this overall return to homeostasis, segregating MHC II^+^ CD38^+^ pro-inflammatory IMs by TB disease state revealed a significantly higher proportion of these cells in mice that relapsed compared to mice that did not relapse or to control mice (Fig. 3D). Both MHC II and CD38 have been identified as biomarkers on IFN-γ^+^ CD4^+^ T cells that discriminate between active TB and LTBI in two cohorts (Balcells et al, 2019; Dutta et al, 2014; Flynn et al, 2011; McCaffrey et al, 2022; Silva Miranda et al, 2012). Future studies should investigate whether they similarly serve as biomarkers on IMs.

Cytokine analysis 3 months post-depletion revealed that G-CSF, GM-CSF, IL-3, IL-12p40, IL-13, IL-17A, and KC were elevated in lungs of mice that relapsed compared to both non-relapsed and control mice (Fig. 3E), while all other analyzed cytokines showed comparable levels across treatment groups (Fig. EV3B). The elevated cytokines promote macrophage (GM-CSF, IL-12p40) and neutrophil (G-CSF, GM-CSF, IL-17A, KC) recruitment and activation (Bajrami et al, 2016; Castellani et al, 2019; De Filippo et al, 2008; Griffin et al, 2012; Ha et al, 1999; Kumar et al, 2022; Lee et al, 2020; Ritzman et al, 2010). Elevated IL-12p40 and IL-17A without an increase in IL-12p70 reflect a shift from Th1 toward Th17 responses (Khader and Cooper, 2008; Lyakh et al, 2008). These findings indicate that Mtb reactivation is associated with reprogramming of the pulmonary cytokine milieu towards a pro-inflammatory state.

### Spleen pathology in mice treated with clodronate liposomes does not correlate with Mtb burden or TB relapse

**Figure 4:**
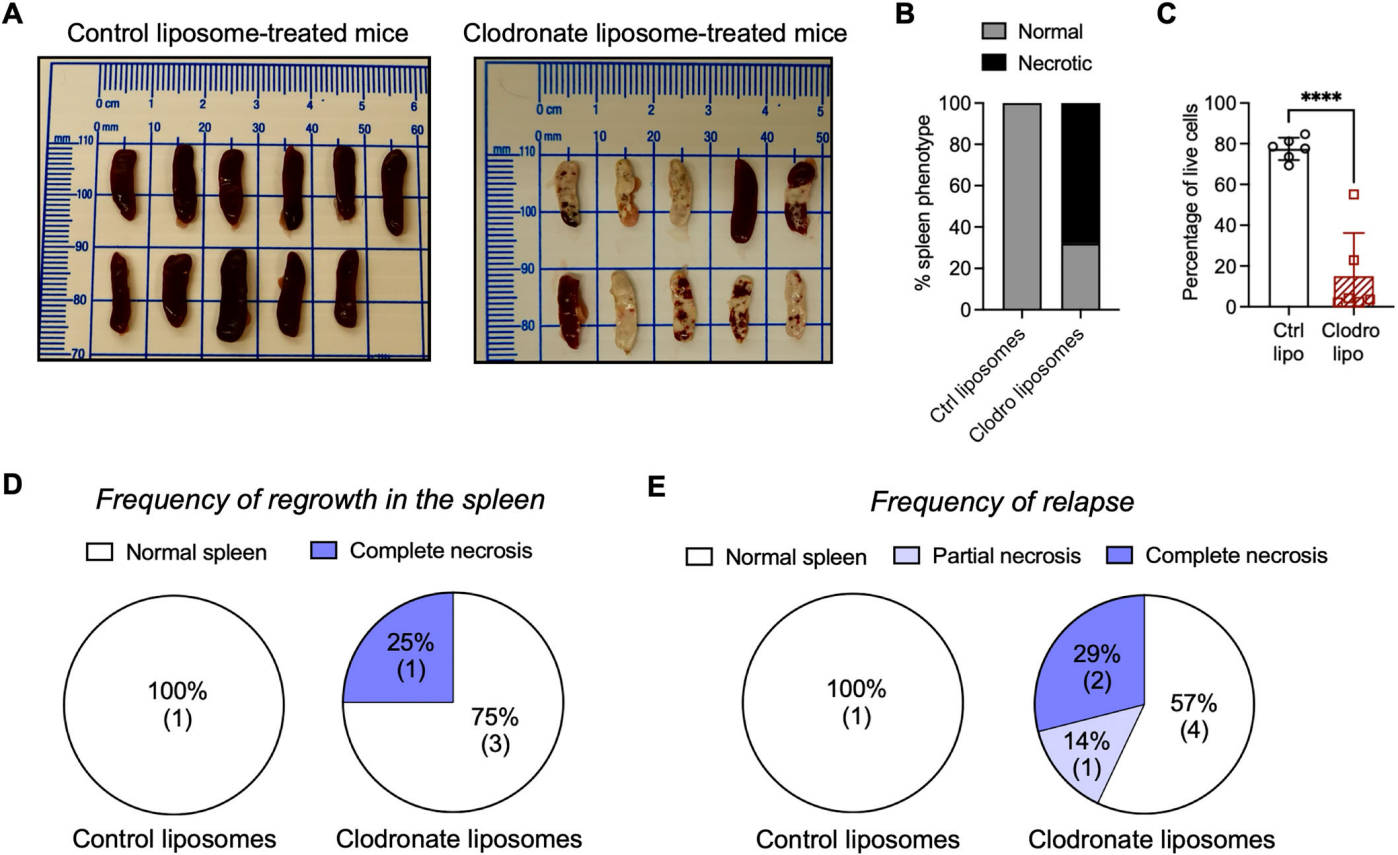
Spleen pathology in mice treated with clodronate liposomes does not correlate with Mtb bacterial burden or TB relapse. (**A**) Gross pathology of spleens harvested from control liposome- or clodronate liposome-treated mice 3 months post-depletion. (**B**) Percentage of spleens that appear necrotic compared to normal in the two treatment groups. (**C**) Flow cytometry was performed with splenocytes isolated from control liposome-treated (Ctrl lipo) and clodronate liposome-treated (Clodro lipo) mice at endpoint, and the percentage of live cells was assessed. Data represent the mean ± SD of 6 biological replicates. ****P < 0.0001 based on an unpaired *t* test. (**D**, **E**) Frequency of Mtb regrowth in the spleen (**D**) and frequency of relapse (**E**) of control liposome- and clodronate liposomes-treated mice segregated by degree of necrosis of the spleen. The number in brackets indicates the number of mice. Exact *P* values are described in Appendix Fig. S4. Source data are available online for this figure.

Unexpectedly, at the study endpoint, most spleens from clodronate liposome-treated mice appeared fibrotic or necrotic, with white lesions partially or completely penetrating the spleen (Fig. 4A). This pathology was observed in 68% of clodronate liposome-treated mice compared to 0% in control mice (Fig. 4B). These affected spleens were rigid and difficult to homogenize, and most splenocytes collected from them were dead, as assessed by flow cytometry (Fig. 4C). As a result, immunoprofiling of splenocytes from these mice was not feasible at this timepoint. Importantly, we found no correlation between Mtb burden in the spleen and the extent of necrosis (Fig. 4D), nor between TB relapse status and the extent of splenic necrosis (Fig. 4E). Thus, while most mice treated with clodronate liposomes developed splenic necrosis, this pathology did not correlate with Mtb regrowth in the spleen or TB relapse.

### Conclusions

Collectively, these findings establish IMs and RMs as indispensable for Mtb control during latent tuberculosis infection in a paucibacillary mouse model. We show that the pulmonary myeloid landscape undergoes dynamic remodeling over the course of infection, with RMs becoming the dominant population during latency and likely sustaining the IM niche through continuous recruitment. A persistent low-grade cytokine milieu, marked by sustained IL-6, TNF, and IL-10, reflects an immunological equilibrium required for bacterial containment. Transient depletion of IMs and RMs disrupts this balance, triggering compensatory neutrophil recruitment and promoting TB relapse.

## Methods

**Table:** Reagents and tools table

Reagent/resource	Reference or source	Identifier or catalog number
**Experimental models**		
C57BL/6 J (*M. musculus*)	The Jackson Laboratory	RRID: IMSR_JAX:000664
*Mycobacterium tuberculosis* H37Rv BPL-DUC	This group	Su et al, 2021
**Antibodies**		Cat, clone, host species raised
Purified rat anti-mouse CD16/32	BioLegend	Clone 93, Cat #101302
AF700 rat anti-mouse CD45	BioLegend	Clone 30-F11, Cat #103128
BUV395 rat anti-mouse CD11b	BD Biosciences	Clone M1/70, Cat #563553
FITC Armenian hamster anti-mouse CD11c	BioLegend	Clone N418, Cat #117306
BV785 rat anti-mouse Ly6C	BioLegend	Clone HK1.4, Cat #128041
APC rat anti-mouse Ly6G	BioLegend	Clone 1A8, Cat #127614
BUV737 rat anti-mouse CD62L	BD Biosciences	Clone MEL-14, Cat #612833
PE/Cy7 rat anti-mouse CD63	BioLegend	Clone NVG-2, Cat #143909
BV421 rat anti-mouse CD170 (Siglec-F)	BioLegend	Clone S17007L, Cat #155509
PE mouse anti-mouse CX3CR1	BioLegend	Clone SA011F11, Cat #149005
BB700 rat anti-mouse I-A/I-E	BD Biosciences	Clone 2G9, Cat #746086
PE/Dazzle594 rat anti-mouse CD38	BioLegend	Clone 90, Cat #102729
BV711 rat anti-mouse CD103	BD Biosciences	Clone M290, Cat #564320
BV650 rat anti-mouse CD24	BD Biosciences	Clone M1/69, Cat #563545
**Chemicals, enzymes, and other reagents**		
Doxycycline chow 2000 ppm	Research Diets, Inc.	Cat #11300-2000i
Control liposomes	LIPOSOMA	Cat #P-030
Clodronate liposomes	LIPOSOMA	Cat #P-040
LIVE/DEAD Fixable Blue Dead Cell Stain Kit, for UV excitation	Thermo Fisher Scientific	Cat #L23105
CellROX Green	Thermo Fisher Scientific	Cat #C10444
**Software**		
FlowJo v10	BD Biosciences	
Belysa v1.2.1	Millipore	
**Other**		
Inhalation Exposure System floor model	Glas-Col	
FACSymphony A5 cytometer	BD Biosciences	
gentleMACS dissociator	Miltenyi Biotec	Cat # 130-093-235
gentleMACS C-tubes	Miltenyi Biotec	Cat # 130-096-334
Microvette 500 K2 anti-coagulant EDTA tubes	Sarstedt	Cat #20.1339.100
Bio-Plex Pro Mouse Cytokine 23-Plex Assay	Bio-Rad	Cat #M60009RDPD
Luminex FLEXMAP 3D System	Millipore	

### Mtb strains and culturing conditions

Mtb H37Rv BPL-DUC has been previously described (Su et al, 2021; Tiwari et al, 2018). Strains were cultured in liquid Middlebrook 7H9 medium supplemented with 0.2% glycerol, 0.05% tyloxapol, and ADNaCl (0.5% BSA, 0.2% dextrose, and 0.085% NaCl), and on Middlebrook 7H10 agar plates supplemented with 0.2% glycerol and Middlebrook OADC enrichment. Antibiotics were added to select for the genetically modified strain, as described (Su et al, 2021).

### Mouse infections

Eight to ten-week-old female C57BL/6 mice were infected with Mtb BPL-DUC using an inhalation exposure system (Glas-Col) with Mtb in a mid-log growth phase to deliver ~100 bacilli per mouse. Mice received doxy-containing chow (2000 ppm, Research Diets, Inc.) for the indicated period. Animals were housed in a biosafety level 3 (BSL3) facility and fed water and chow *ad libitum*. All procedures involving animals were reviewed and approved by the Weill Cornell Medicine Institutional Animal Care and Use Committee (IACUC).

### Paucibacillary mouse model of latent tuberculosis infection

We employed an inducible paucibacillary mouse model of latent tuberculosis infection (Su et al, 2021). This model utilizes Mtb BPL-DUC, an engineered strain that allows inducible depletion of the essential biotin protein ligase (BPL) at both the transcriptional and translational levels, resulting in bacterial death (Tiwari et al, 2018). Mice infected by aerosol with BPL-DUC were given doxycycline (doxy)-containing chow starting on day 28 post-infection to deplete BPL. Since BPL inactivation inhibits all lipid biosynthesis, the bacteria are killed, and the infection is apparently sterilized. Doxy treatment was discontinued on day 140, 4 weeks after no Mtb could be recovered from mouse organs, allowing BPL expression to resume in any surviving, non-culturable bacteria. Several months after cessation of doxy administration, persisting Mtb populations reactivate, causing TB relapse.

### Enumeration of bacterial burden

To enumerate CFUs, organs were homogenized in PBS and cultured on 7H10 agar. To recover Mtb BPL-DUC from mice that were treated with doxycycline, charcoal (0.4% wt/vol) was added to the plates. Agar plates were incubated for at least 4 weeks at 37 °C.

### Depletion of interstitial macrophages

Control liposomes (PBS-loaded) and clodronate-loaded liposomes were purchased from LIPOSOMA research. To deplete interstitial macrophages, mice were injected intravenously with 200 μL clodronate liposomes during the paucibacillary phase every 2–3 days over a period of 2 weeks for a total of four injections. PBS-loaded liposomes were used as controls. The efficiency of macrophage depletion was assessed by flow cytometry.

### Flow cytometry

For flow cytometry, mouse lungs and spleens were isolated as previously described (Su et al, 2021), with some modifications. Briefly, lungs were isolated in digestion media containing 0.5% BSA, 150 U/mL collagenase IV, and 100 μg/mL DNase I enzymes in C-tubes (Miltenyi Biotec). Lungs were homogenized using the gentleMACS Tissue Dissociator using the program *m_lung_02.01* and then incubated at 37 °C for 45 min. Cells were homogenized one more time using the program *m_lung_01_02* and filtered using 70-μm cell strainers, collected by centrifugation at 500x*g* for 5 min, and resuspended in 3 mL red blood cell lysis buffer (eBiosciences). Cells were incubated for 5 min at room temperature with occasional shaking. PBS was added to the tube, centrifuged, and resuspended in RPMI 1640 media. Spleens were harvested in PBS in C-tubes and homogenized using the gentleMACS Tissue Dissociator. Cells were filtered using 40-μm cell strainers, collected by centrifugation at 500×*g* for 5 min, and resuspended in 3 mL red blood cell lysis buffer and processed similarly to lung cells. Samples were seeded in a 96-well plate and kept at 4 °C overnight. Cells were then stained with Zombie UV LIVE/DEAD Fixable Blue stain (Thermo Fisher Scientific) to discriminate between live and dead cells. Purified anti-CD16/32 antibody (clone 93) was used to block Fc receptors on all cells before staining. The antibody cocktail was prepared in a 3:1 solution of Cell Staining Buffer and Brilliant Stain Buffer, and cells were stained for 30 min at 4 °C with the following anti-mouse antibodies: CD45-AF700 (clone 30-F11), CD11b-BUV395 (clone M1/70), CD11c-FITC (clone N418), Ly6C-BV785 (clone HK1.4), Ly6G-APC (clone 1A8), CD62L-BUV737 (clone MEL-14), CD63-PE-Cy7 (clone NVG-2), Siglec-F-BV421 (clone S17007L), CX3CR1-PE (clone SA011F11), I-A/I-E-BB700 (clone 2G9), CD38-PE/Dazzle594 (clone 90), CD103-BV711 (clone M290) and CD24-BV650 (clone M1/69). The cells were then fixed with fixation buffer (BioLegend) for 30 min at room temperature and washed twice with cell staining buffer before being taken out of the BSL3. Samples were acquired using the BD FACSymphony A5 cytometer and analyzed with FlowJo v10.

### Reactive oxygen species assay

To measure the levels of reactive oxygen species (ROS) in mice, blood was collected from the tail vein of mice into an anti-coagulant EDTA tube. Cells were transferred to a round-bottom 96-well plate, centrifuged at 500×*g* for 5 min, and resuspended in ROS media (RPMI plus 10% FBS, 1 mM MgCl_2_, and 1 mM CaCl_2_). Cells were stimulated ex vivo with 100 ng/mL PMA at 37 °C for 30 min. In all, 5 μM CellROX Green (Thermo Fisher Scientific) was added to each sample, and cells were incubated at 37 °C for 30 min, after which cells were centrifuged and washed three times in PBS. Fc block was diluted in FACS-ROS buffer (PBS plus 2% heat-inactivated FBS and 2 mM EDTA) and added to all samples, and cells were incubated at 4 °C for 10 min. After centrifugation and removing the supernatant, cells were incubated with an antibody cocktail prepared in FACS-ROS buffer with antibodies CD45-AF700, CD11b-BUV395, and Ly6G-APC to identify neutrophils. The rest of the flow cytometry staining was performed as aforementioned. Before acquiring the samples on a BD FACSymphony A5 cytometer, cells were resuspended in 200 μL FACS-ROS buffer.

### Multiplex cytokine analysis

The concentrations of various cytokines and chemokines in the lung homogenates were measured using the Bio-Plex Pro Mouse Cytokine Group I 23-plex Assay (Bio-Rad) according to the manufacturer’s protocol and as described previously (Tsai et al, 2024). Briefly, 50 μL of coupled beads were added to each well of a black, 96-well flat-bottom plate and washed twice with 100 µL of Bio-Plex wash buffer. Samples were diluted 1:2 in Bio-Plex sample diluent containing 0.5% (w/v) bovine serum albumin. Next, 50 µL of diluted samples, standards, and blanks (diluent + BSA) were added to the wells and incubated at room temperature for 30 min with shaking (850 rpm) in the dark. Plates were washed three times and incubated with 25 µL of 1X detection antibodies for 30 min with shaking in the dark. After another three washes, 50 µL of 1× Streptavidin-PE was added and incubated at room temperature for 10 min with shaking in the dark. Following three final washes, beads were resuspended in 125 µL of Bio-Plex assay buffer and analyzed using FLEXMAP 3D system (Millipore) according to the manufacturer’s instructions. Raw data were processed and analyzed using Belysa software (version 1.2.1, Millipore).

### Statistical analyses

Flow cytometry data were analyzed by *t* test, or by one-way or two-way ANOVA with Tukey’s multiple comparisons test, depending on the experiment.

The cytokine measurements for days 1, 28, and 180 (Fig. 1F) and day 194 (Fig. 2K) were log-transformed to accommodate the wide variation of scales across various cytokines. These were analyzed by two-way ANOVA with a Tukey’s multiple comparisons test. Cytokine measurements for endpoint at day 294 (Fig. 3D) were analyzed by fitting a linear-mixed model with random effects and applying an *F* test to evaluate the significance of coefficients in the model. 11 cytokines (Cyt) were included, and three treatment groups (Tx) were used: control liposomes, clodronate-treated/non-relapsed, and clodronate-treated/relapsed. The Tx*Cyt interaction term incorporated distinct means for each cytokine in each treatment group. Since 4 of the 22 mice had relapsed, resulting in higher and more variable measurements, the relapsed mice were indicated with a binary covariate (Rpd) and the coefficients were treated as random effects for each mouse, allowing for different magnitudes of increase for each mouse. The data for all cytokines, mice, and controls were fit using *lmer()* in R, using the following formula: *log(obs)* ~ Tx*Cyt + *(Rpd|Mouse)*. After fitting the model, all three pairwise contrasts among treatments were compared for each cytokine using an *F* test using *emmeans()* in R. *P* values were adjusted for multiple comparisons using Tukey’s method (Tukey, 1949). Cytokines exhibiting significant differences (*P* value < 0.05) between clodronate/relapsed and control, or clodronate/relapsed and clodronate/non-relapsed, were identified.

The Poisson model was used for evaluating differences in reactivation rates (Fig. 3A). To estimate the reactivation rates for each treatment, data from mice at multiple timepoints were combined by summing the number of mice at each timepoint weighted by the number of days, i.e., “mouse-days”. Data was combined over two independent experiments. We used a Poisson test to quantify the statistical significance of differences among these rates of reactivation. Specifically, we applied an exact test of the ratio of Poisson rate parameters (Gu et al, 2008), as implemented in the *poisson.test()* function in R. Samples were blinded for analysis. Mice that were sick from a non-TB infection, or for which there were technical issues with the sample, were excluded from the analysis.

### Graphics

Synopsis graphics were created with bioRender.com and smart.servier.com.

## Supplementary information

Appendix

Peer Review File

Source data Fig. 1

Source data Fig. 2

Source data Fig. 3

Source data Fig. 4

Figure EV2 Source Data

Figure EV3 Source Data

Expanded View Figures

## Data Availability

This study includes no data deposited in external repositories. The source data of this paper are collected in the following database record: biostudies:S-SCDT-10_1038-S44321-026-00432-6.
