# Supplementary material for: Interstitial and recruited macrophages prevent tuberculosis relapse by limiting immune evasion
Source: EMBO Mol Med. 2026 Apr 29;18(6):2021–37. doi: 10.1038/s44321-026-00432-6 (PMC13270126; doi:10.1038/s44321-026-00432-6)
Supplement: Supplementary file 1 — Appendix [file 44321_2026_432_MOESM1_ESM.pdf]

APPENDIX

Interstitial and recruited macrophages prevent tuberculosis relapse by limiting immune evasion

Valerie Vinette[1], Anthony Castro[1], Heather Kim[1], Carolina Trujillo[1], Min Xie[2], Martin Gengenbacher[2,3], Thomas R. Ioerger[4], Sabine Ehrt[1]

Table of Contents

Appendix Figure S1. The pulmonary myeloid cell compartment and cytokine milieu are altered in a paucibacillary mouse model of latent tuberculosis infection (Statistics for Figure 1)……………..…………………………………………..………………………………..2

Appendix Figure S2. Clodronate liposome treatment during LTBI leads to a depletion of interstitial and recruited macrophages and increased recruitment of neutrophils to the lung (Statistics for Figure 2)……………..……………………………………..…………………..3

Appendix Figure S3. Depletion of interstitial and recruited macrophages during latency results in increased tuberculosis relapse in mice, accompanied by elevated pro-inflammatory interstitial macrophages and cytokines in the lung (Statistics for Figure 3). ……………………………..……………………………………..………………………………..4

Appendix Figure S4. Spleen pathology in mice treated with clodronate liposomes does not correlate with Mtb bacterial burden or TB relapse (Statistics for Figure 4).……..……..5

Appendix Figure S5. Immunophenotype of dendritic cells and eosinophils in the lung and spleen upon interstitial and recruited macrophage depletion during LTBI (Statistics for EV Figure 2)………………………………………………………………………………..6

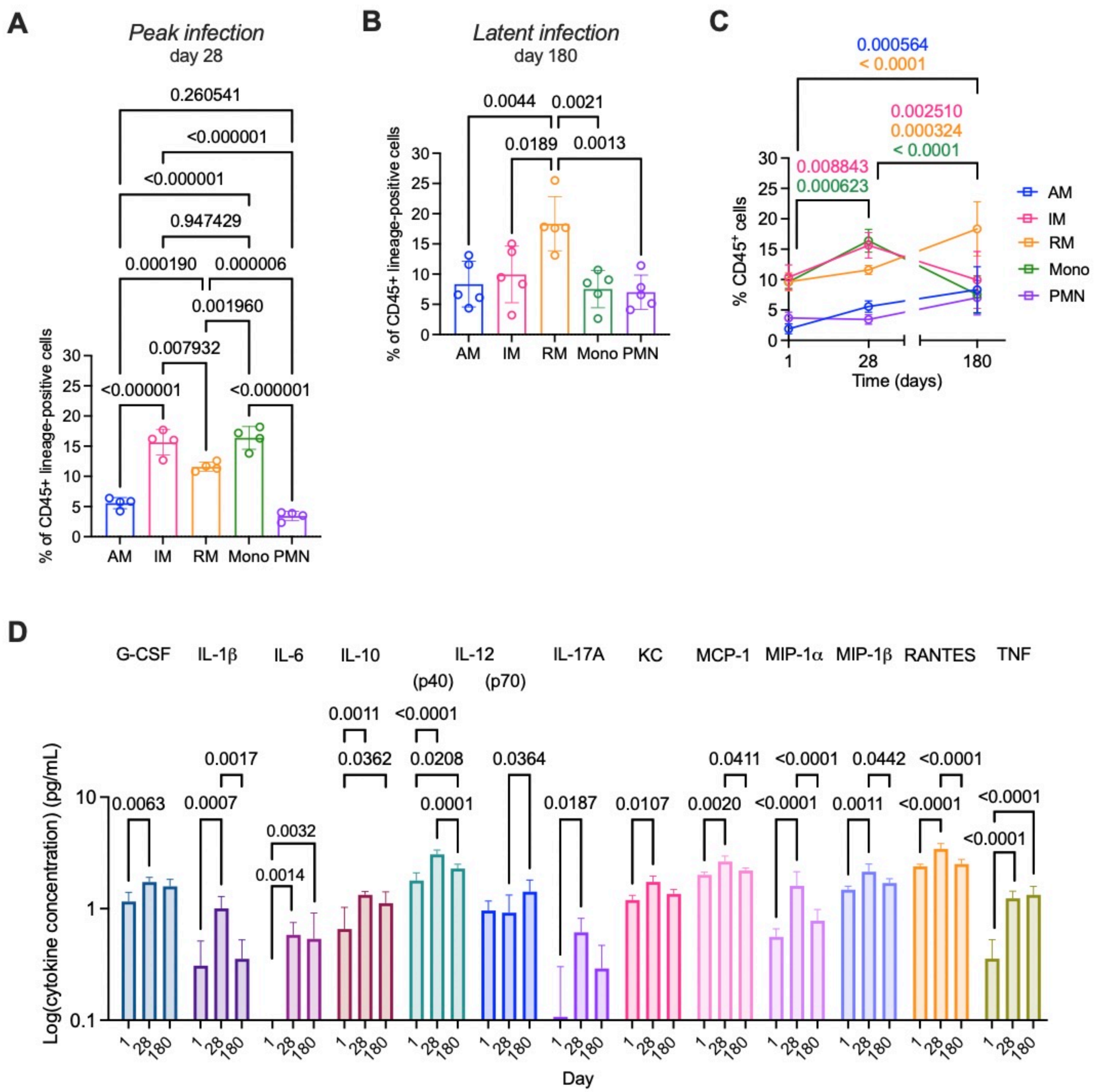

Appendix Figure S1 – The pulmonary myeloid cell compartment and cytokine milieu are altered in a paucibacillary mouse model of latent tuberculosis infection (Statistics for Figure 1). (A-C) Immunoprofiling was performed by flow cytometry with lung homogenates to assess the frequency of alveolar macrophages (AM), interstitial macrophages (IM) recruited macrophages (RM), monocytes (Mono) and neutrophils (PMN) at the peak of infection (A) and during the paucibacillary phase, prior to treatment with liposomes (B). Data represent the mean ± SD of four (A) or five (B) biological replicates. (C) Frequency of macrophage, monocyte and neutrophil populations in the lung over time before treatment with liposomes. Data represent the mean ± SD of four (day 1, 28) or five (day 180) biological replicates. (D) Cytokine concentrations in the lung of mice at days 1, 28 and 180. Data represent the mean ± SD of four biological replicates. P values based on one-way ANOVA (for A-B) or two-way ANOVA (for C-D) statistical test with Tukey's multiple comparisons tests

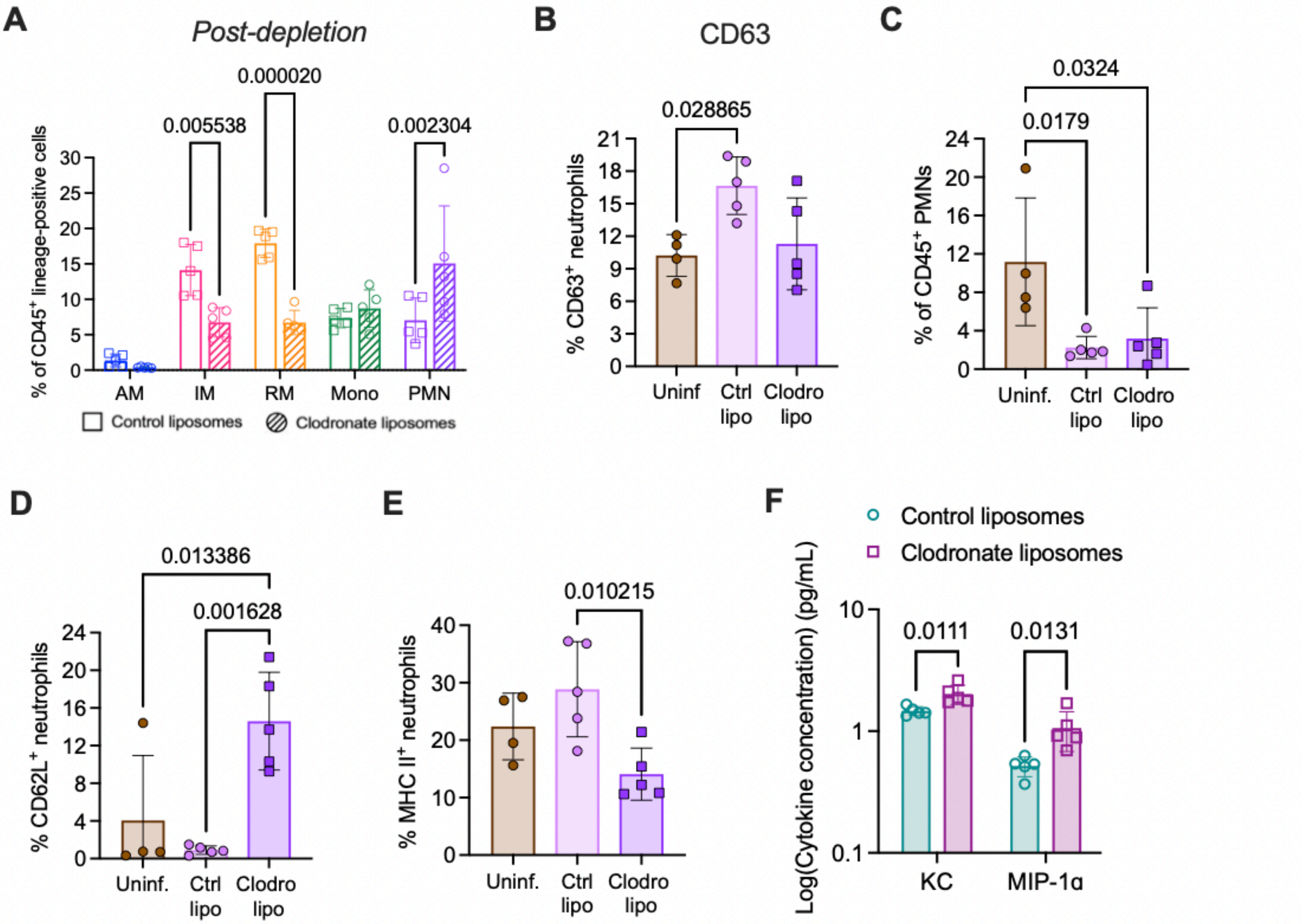

Appendix Figure S2 – Clodronate liposome treatment during LTBI leads to a depletion of interstitial and recruited macrophages and increased recruitment of neutrophils to the lung (Statistics for Figure 2). Mice were treated with control or clodronate liposomes for two weeks during the paucibacillary phase, and lung (A-B, F) and spleen (C-E) were analyzed. (A-B) Immunoprofiling was performed by flow cytometry with lung homogenates to assess the frequency of alveolar macrophages (AM), interstitial macrophages (IM), recruited macrophages (RM), monocytes (Mono) and neutrophils (PMN) immediately post-depletion with liposomes (A). Data represent the mean ± SD of five biological replicates. (B) Flow cytometry from lung homogenates following liposome treatment was done to assess the frequency of neutrophils expressing the degranulation marker CD63 in uninfected mice (Uninf) and mice treated with control liposomes (Ctrl lipo) or clodronate liposomes (Clodro lipo). (C-E) Flow cytometry from spleen homogenates following liposome treatment was performed to assess the frequency of neutrophils (C) and the fraction of these neutrophils expressing the activation markers CD62L (D) or MHC II (E). For B-E, data represent the mean ± SD of four (uninfected) or five (control or clodronate liposomes) biological replicates. (F) Quantification of cytokine concentrations in the lung of mice treated with control or clodronate liposomes immediately following liposome treatment. Data represent the mean ± SD of five biological replicates. P values based on two-way ANOVA statistical test with Tukey's (for A) or Šídák's (for F) multiple comparisons test, or on one-way ANOVA statistical test with Tukey's multiple comparisons test (for B-E).

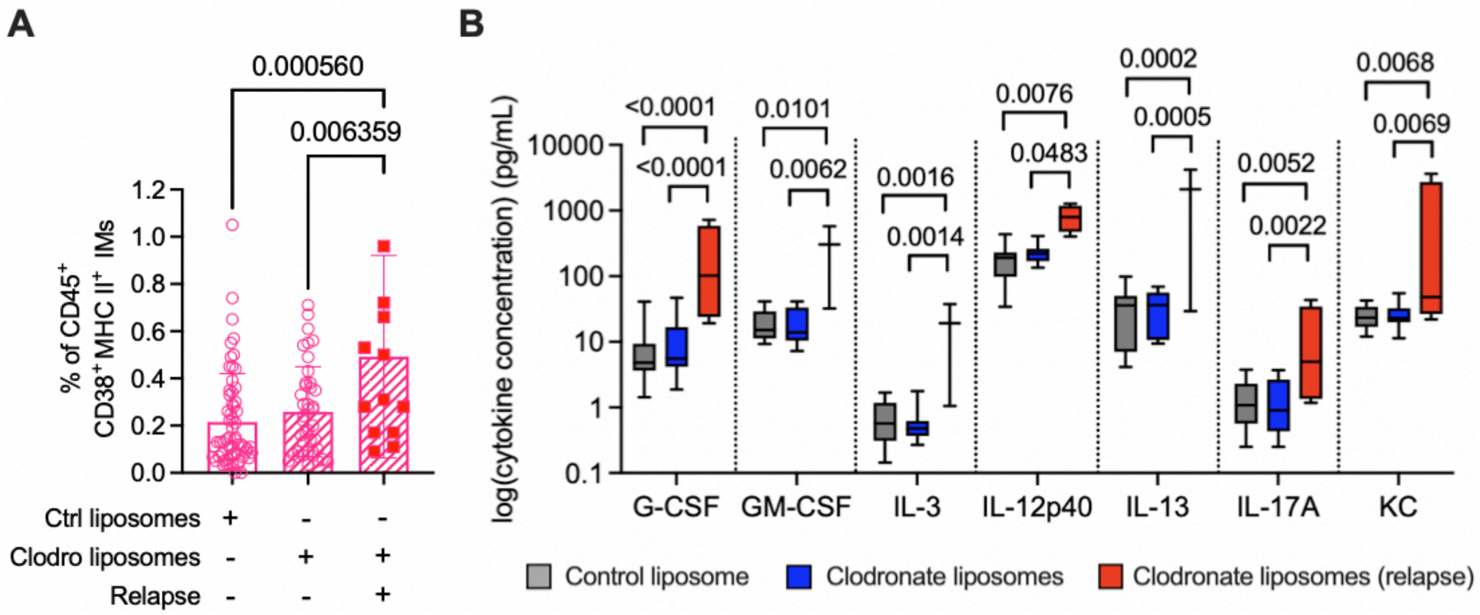

Appendix Figure S3 – Depletion of interstitial and recruited macrophages during latency results in increased tuberculosis relapse in mice, accompanied by elevated pro-inflammatory interstitial macrophages and cytokines in the lung (Statistics for Figure 3). (A) Frequency of pro-inflammatory IMs (CD38$^+$ MHC II$^+$ IMs) present in the lung of treated mice three-months post-depletion as assessed by flow cytometry. Data represent the mean ± SD of 61 (control liposomes), 41 (clodronate liposomes, no relapse) or 13 (clodronate liposomes, relapse) biological replicates. P values based on two-way ANOVA statistical test with Tukey's multiple comparisons test. (B) Cytokine concentrations in lung homogenates from mice treated with control liposomes (gray, n = 22), or from clodronate liposome-treated mice that relapsed (red, n = 4) or did not relapse (blue, n = 14). Data represent the mean ± SD of biological replicates. P values based on an F-test. Box plots depict the interquartile range (IQR), extending from the 25th to the 75th percentiles of the data, and the central line in the middle of each box indicates the median value. Whiskers extend the full range of the data (min to max).

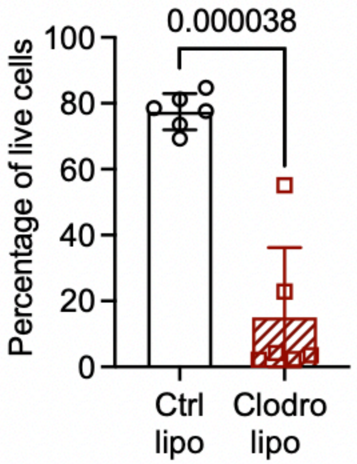

Appendix Figure S4 – Spleen pathology in mice treated with clodronate liposomes does not correlate with Mtb bacterial burden or TB relapse (Statistics for Figure 4). Flow cytometry was performed with splenocytes isolated from control liposome-treated (Ctrl lipo) and clodronate liposome-treated (Clodro lipo) mice at endpoint and the percentage of live cells was assessed. Data represent the mean ± SD of 6 biological replicates. P value based on an unpaired t test.

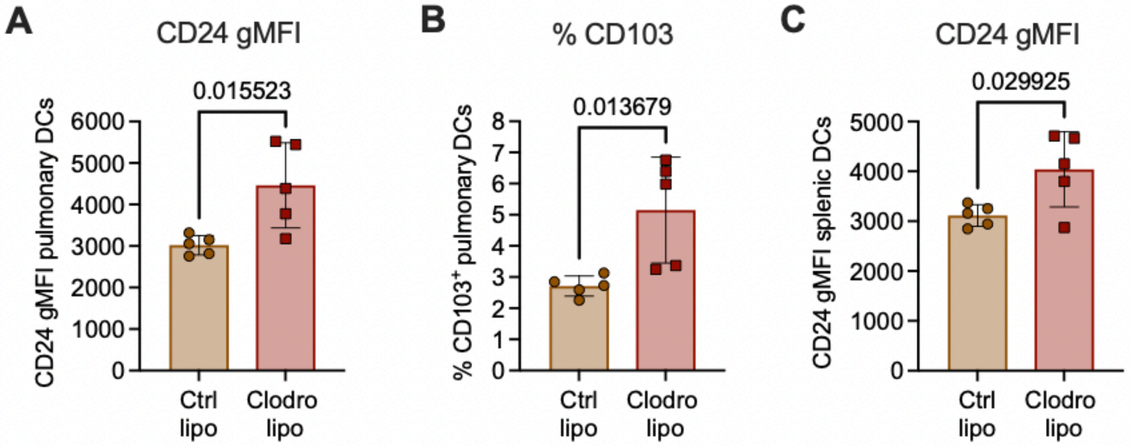

Appendix Figure S5 – Immunophenotype of dendritic cells and eosinophils in the lung and spleen upon interstitial and recruited macrophage depletion during LTBI (Statistics for EV Figure 2). Lung and spleen were harvested from mice treated with control or clodronate liposomes immediately following depletion of interstitial macrophages. Immunoprofiling was performed by flow cytometry to assess pulmonary (A-B) and splenic (C) $CD11c^+$ DCs. The gMFI of CD24 (A) and frequency of $CD103^+$ (B) pulmonary $CD11c^+$ DCs are displayed. (C) gMFI of CD24 splenic $CD11c^+$ DCs. Data represent mean ± SD of 5 biological replicates. P values based on an unpaired t test.
