# Supplementary figures and images for: Interstitial and recruited macrophages prevent tuberculosis relapse by limiting immune evasion
Source: EMBO Mol Med. 2026 Apr 29;18(6):2021–37. doi: 10.1038/s44321-026-00432-6 (PMC13270126; doi:10.1038/s44321-026-00432-6)

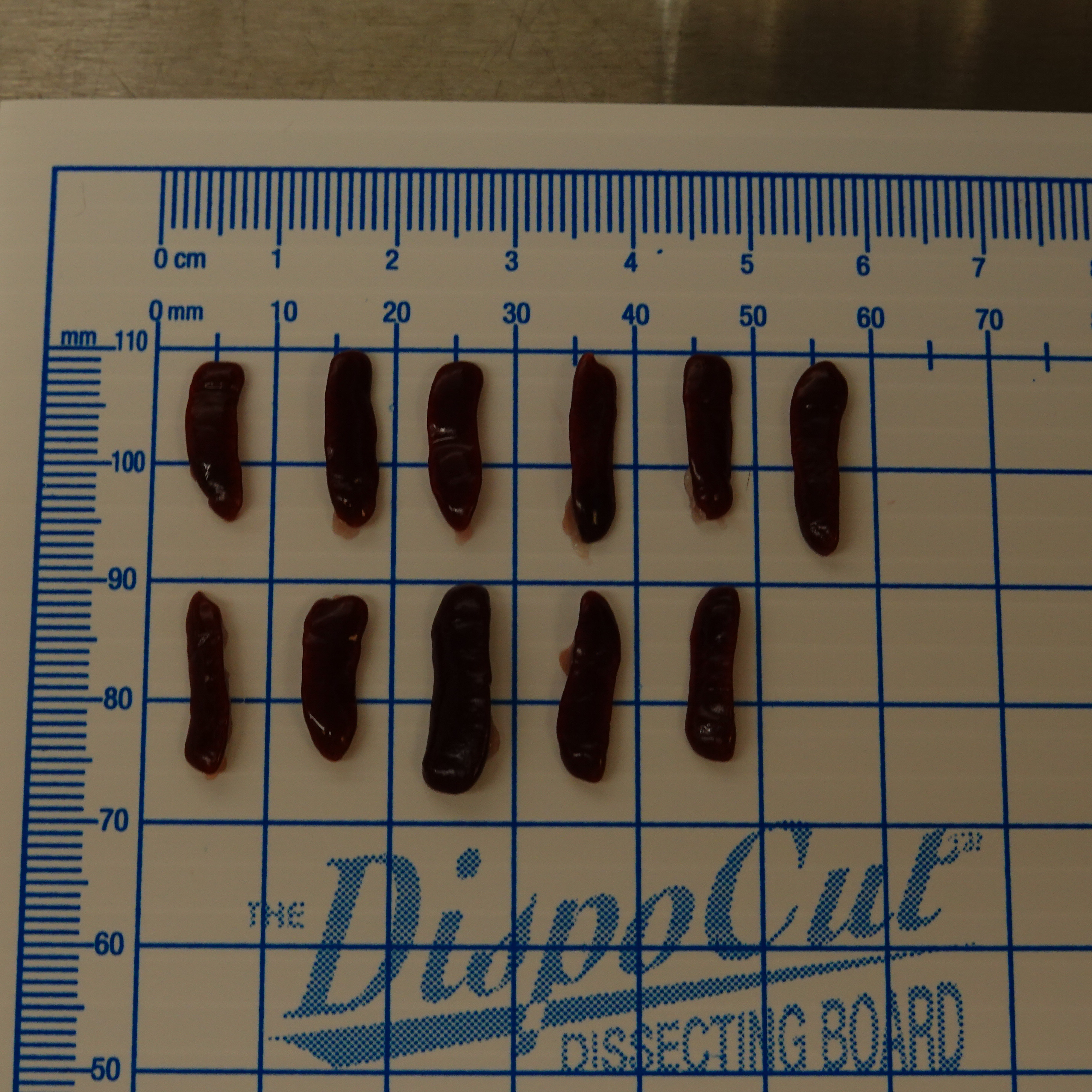

Supplement: Supplementary file 6 — Source data Fig. 4 [file 44321_2026_432_MOESM6_ESM.zip › Figure 4/Figure 4A/Control liposomes.tiff]

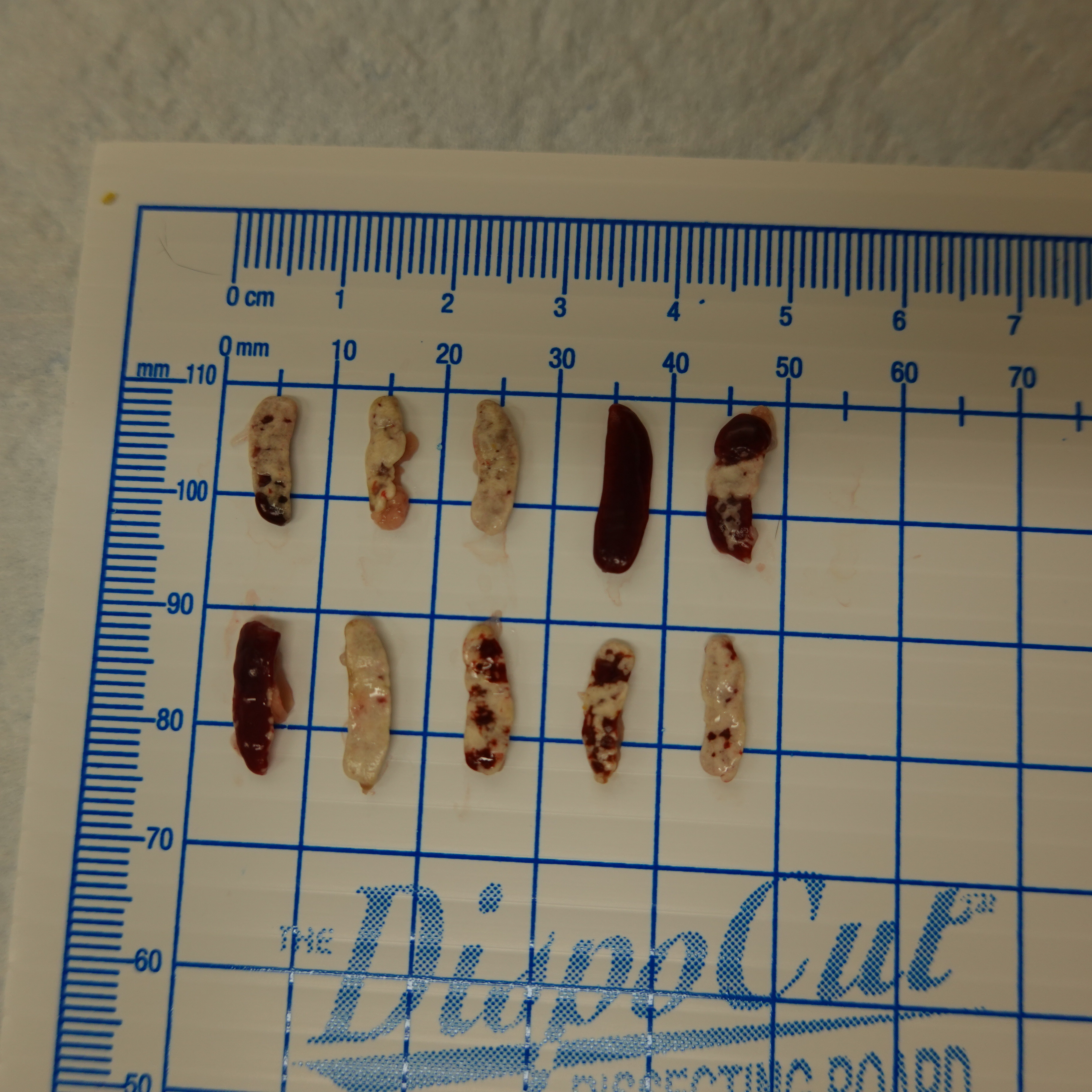

Supplement: Supplementary file 6 — Source data Fig. 4 [file 44321_2026_432_MOESM6_ESM.zip › Figure 4/Figure 4A/Clodronate liposomes.tiff]
