# Supplementary material for: Interstitial and recruited macrophages prevent tuberculosis relapse by limiting immune evasion
Source: EMBO Mol Med. 2026 Apr 29;18(6):2021–37. doi: 10.1038/s44321-026-00432-6 (PMC13270126; doi:10.1038/s44321-026-00432-6)
Supplement: Supplementary file 9 — Expanded View Figures [file 44321_2026_432_MOESM9_ESM.pdf]

Expanded View Figures

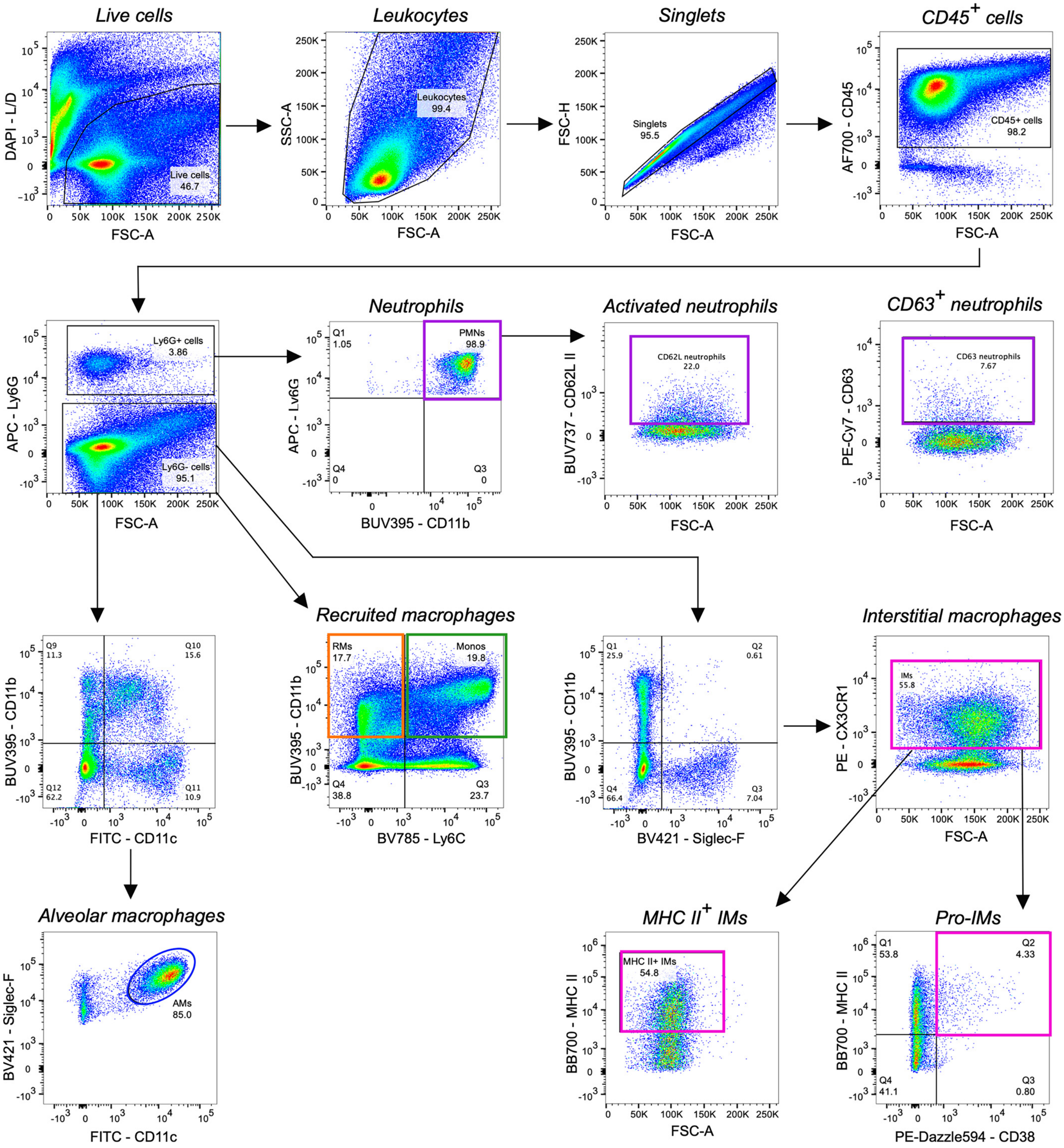

Figure EV1. Gating strategy to identify myeloid cell populations.

Gating strategy to identify neutrophils (PMNs, purple), alveolar macrophages (AMs, blue), recruited macrophages (RMs, orange), monocytes (Monos, green), and interstitial macrophages (IMs, pink) by flow cytometry.

 © The Author(s)

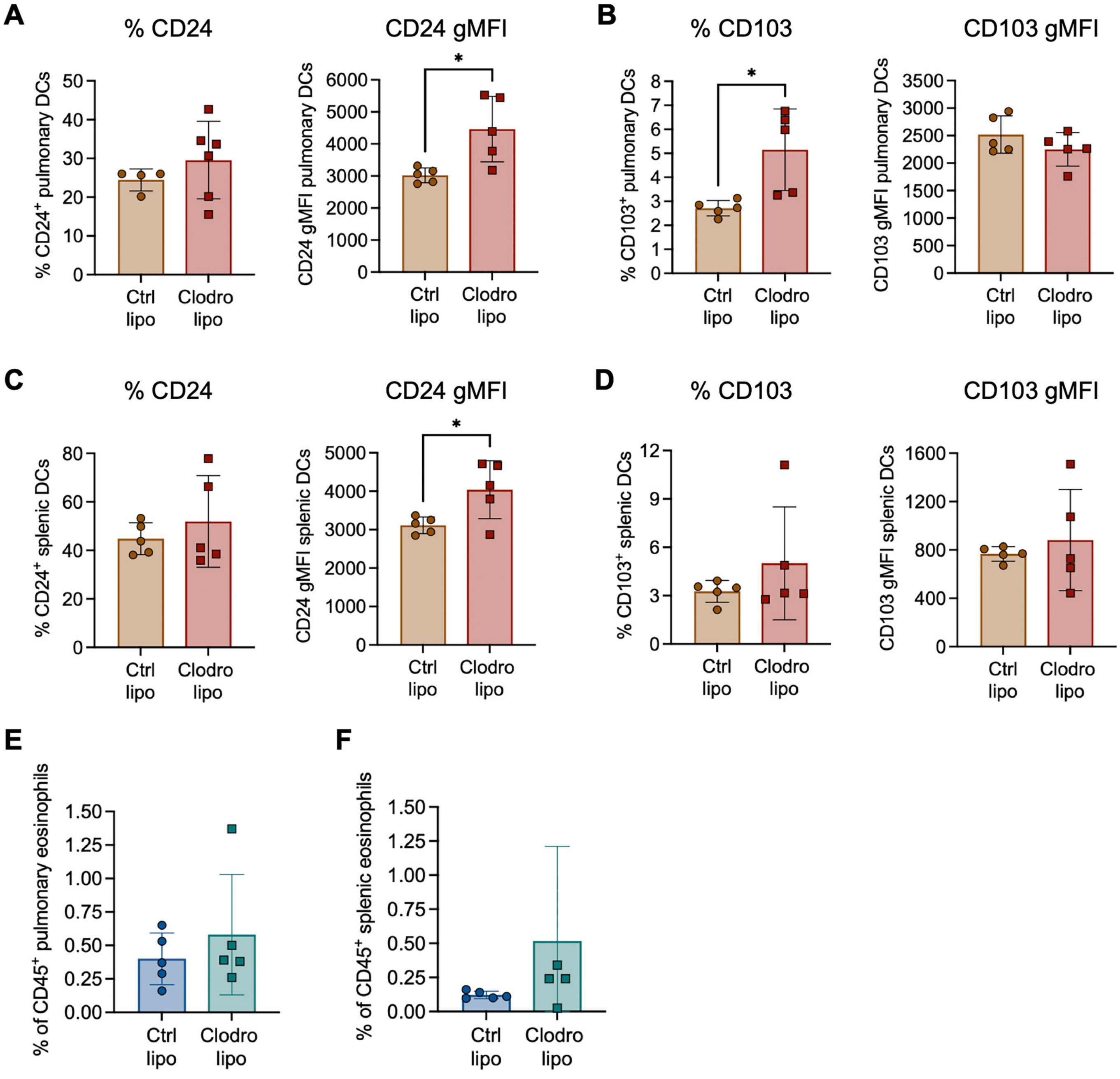

Figure EV2. Immunophenotype of dendritic cells and eosinophils in the lung and spleen upon interstitial and recruited macrophage depletion during LTBI.

Lung and spleen were harvested from mice treated with control or clodronate liposomes immediately following depletion of interstitial macrophages. (**A–D**) Immunoprofiling was performed by flow cytometry to assess pulmonary CD11c$^+$ DCs and their frequency and gMFI of CD24 (**A**) and CD103 (**B**), as well as the frequency of splenic CD11c$^+$ DCs for CD24 (**C**) and CD103 (**D**). (**E**, **F**) Immunoprofiling was performed by flow cytometry to assess the frequency of pulmonary and splenic eosinophils, identified as CD11b$^+$ Siglec-F$^+$ cells. Data represent mean ± SD of five biological replicates. *$P < 0.05$ based on an unpaired *t* test. Exact *P* values are described in Appendix Fig. S5. Source data are available online for this figure.

© The Author(s)

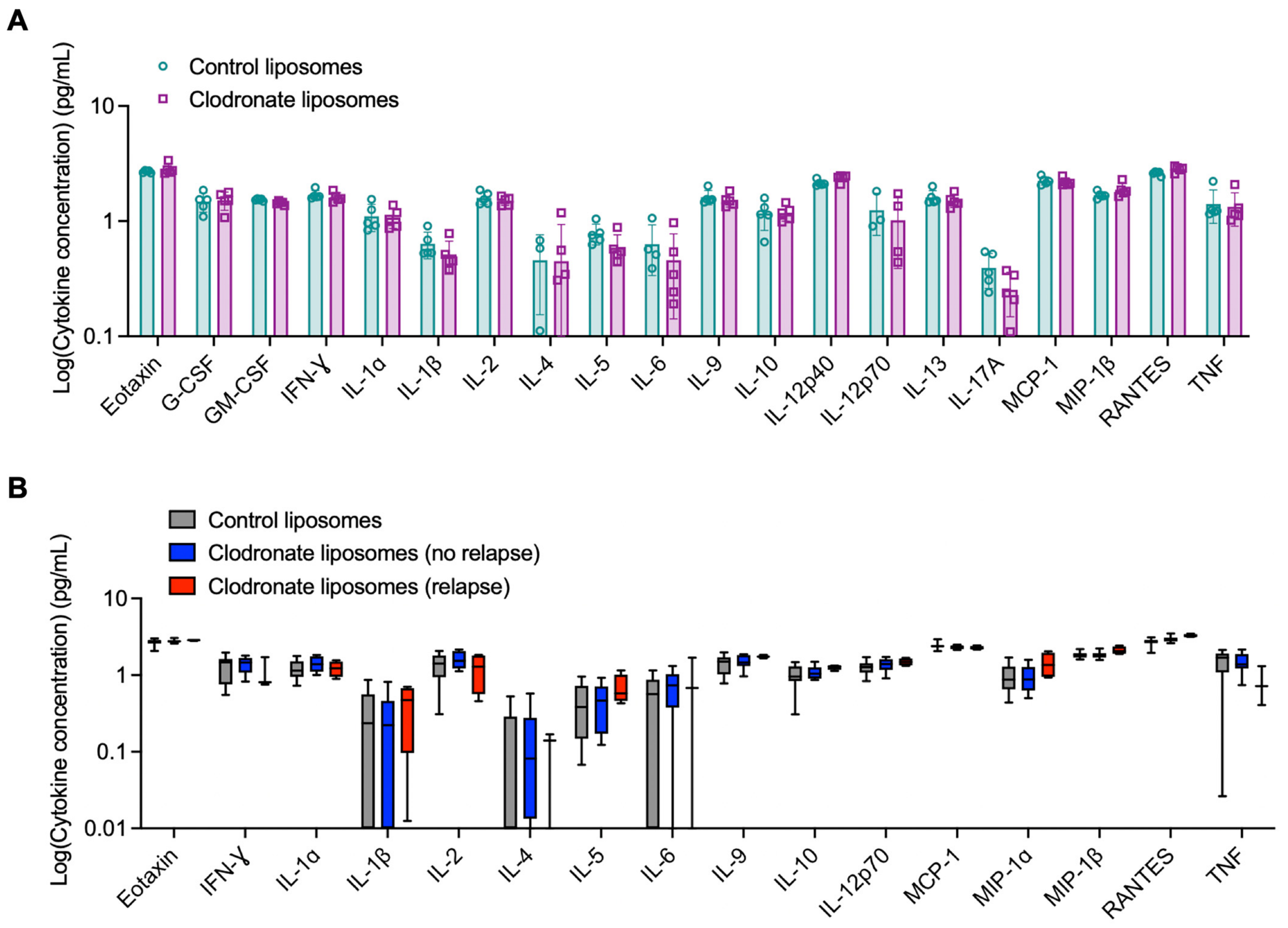

Figure EV3. Pulmonary cytokine levels after treatment with liposomes.

Quantification of cytokine concentrations in the lung of mice treated with control or clodronate liposomes immediately following treatment (**A**) and at experimental endpoint (**B**). For (**A**), data represent the mean ± SD of five biological replicates. Statistical analysis performed by a two-way ANOVA statistical test with Tukey's multiple comparisons test. For (**B**), data represent the mean ± SD of 22 (control liposomes), 14 (clodronate liposomes, no relapse) or 4 (clodronate liposome, relapse) biological replicates. Statistical analysis performed by the *F* test. Box plots depict the interquartile range (IQR), extending from the 25th to the 75th percentiles of the data, and the central line in the middle of each box indicates the median value. Whiskers extend the full range of the data (min to max). Source data are available online for this figure.

 © The Author(s)

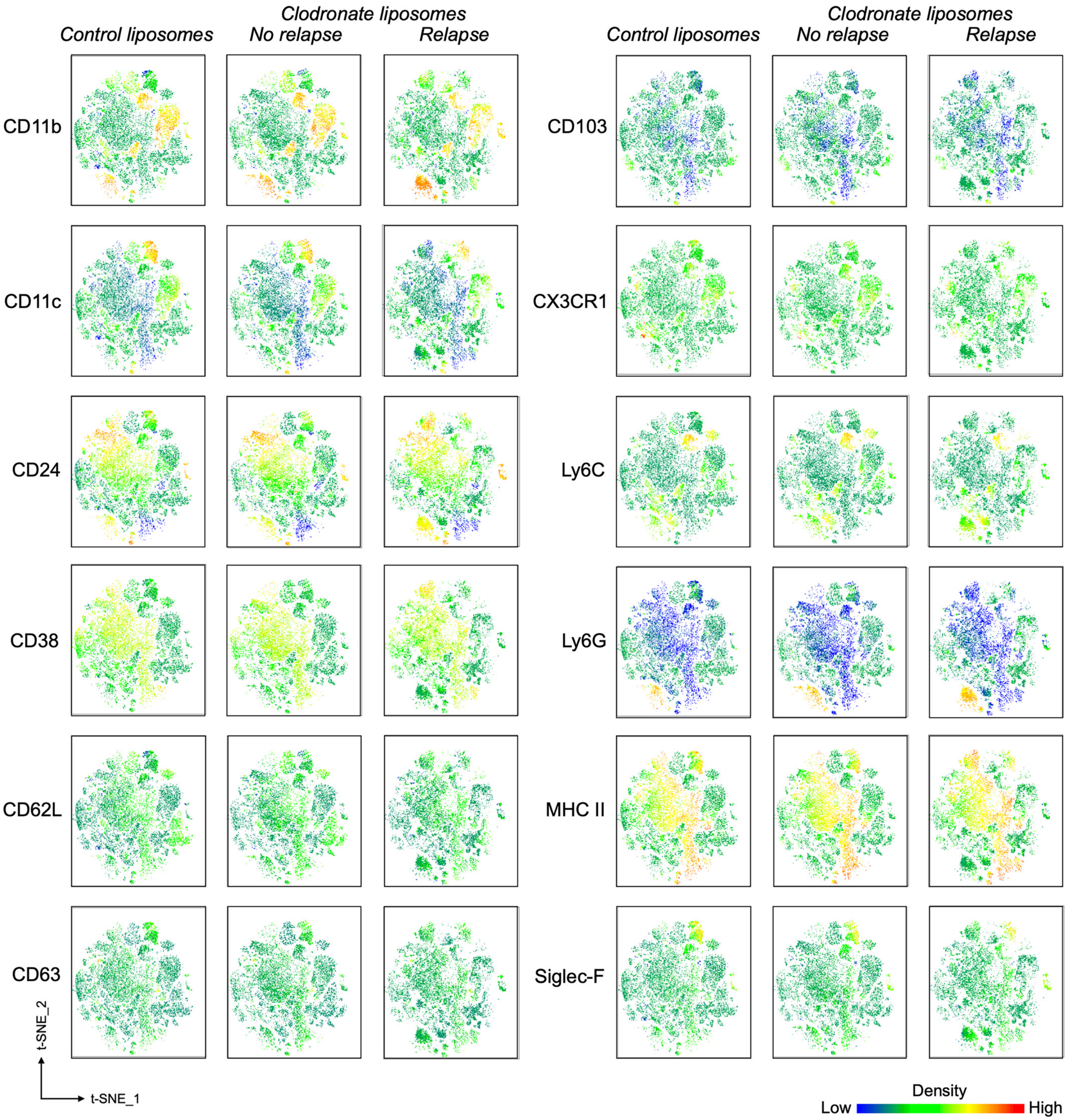

Figure EV4. Cell surface markers are expressed at similar levels at the experimental endpoint.

Multigraph color mapping was performed with flow cytometry data from uninfected mice and infected mice treated with control or clodronate liposomes to visualize the expression of all flow cytometry markers tested across the various t-SNE clusters.
