# Supplementary material for: Interstitial and recruited macrophages prevent tuberculosis relapse by limiting immune evasion
Source: EMBO Mol Med. 2026 Apr 29;18(6):2021–37. doi: 10.1038/s44321-026-00432-6 (PMC13270126; doi:10.1038/s44321-026-00432-6)
Supplement: Supplementary file 2 — Peer Review File [file 44321_2026_432_MOESM2_ESM.pdf]

EMBO Molecular Medicine

Interstitial and recruited macrophages prevent tuberculosis relapse by limiting immune evasion

Valerie Vinette, Anthony Castro, Heather Kim, Carolina Trujillo, Min Xie, Martin Gengenbacher, Thomas Ioerger, and Sabine Ehrt

Corresponding author: Sabine Ehrt (sae2004@med.cornell.edu)

Review Timeline:

Submission Date:	18th Sep 25
Editorial Decision:	14th Oct 25
Appeal:	25th Jan 26
Editorial Decision:	25th Feb 26
Revision Received:	14th Mar 26
Editorial Decision:	26th Mar 26
Revision Received:	9th Apr 26
Accepted:	13th Apr 26

Editor: Zeljko Durdevic

Transaction Report:

(Note: With the exception of the correction of typographical or spelling errors that could be a source of ambiguity, letters and reports are not edited. Depending on transfer agreements, referee reports obtained elsewhere may or may not be included in this compilation. Referee reports are anonymous unless the Referee chooses to sign their reports.)

14th Oct 2025

Dear Prof. Ehrt,

Thank you for the submission of your manuscript to EMBO Molecular Medicine. We have now received feedback from the two reviewers who agreed to evaluate your manuscript. As you will see from their reports pasted below, both referees recognize potential interest of the study but also raise serious concerns particularly regarding, but not limited to, descriptive nature of the study, limited conceptual advance, and several technical shortcomings. Given these considerations, I am afraid that we do not feel it would be productive to call for a revised version of your manuscript at this stage and therefore we cannot offer to publish it.

That being said and given the potential interest of the findings, we would, however, be willing to consider a new manuscript on the same topic if at some time in the near future you obtained data that would considerably strengthen the message of the study and address the referees concerns in full. To be completely clear, however, I would like to stress that if you were to send a new manuscript this would be treated as a new submission rather than a revision and would be reviewed afresh, in particular with respect to the literature and the novelty of your findings at the time of resubmission. If you decide to follow this route, please make sure you nevertheless upload a letter of response to the referees' comments.

I am sorry that I could not bring better news this time and hope that the referee comments are helpful in your continued work in this area.

Yours sincerely,

Zeljko Durdevic

Zeljko Durdevic
Senior Editor
EMBO Molecular Medicine

***** Reviewer's comments *****

Referee #1 (Comments on Novelty/Model System for Author):

The technical quality of this manuscript is high as it includes necessary controls and appropriate statistical methods for data analysis. While the novelty of the study is medium as it is descriptive without a deep mechanistic analysis, it uncovered a previously undefined role of lung interstitial macrophages in controlling TB relapse during latent TB.

Referee #1 (Remarks for Author):

It has been known that different lung macrophage populations have distinct functions at the early stage of Mtb infection. Lung alveolar macrophages (AM) function as a permissive niche facilitating the growth and dissemination of Mtb whereas interstitial macrophages (IM) are more restrictive for Mtb during the first two weeks of infection. This study took one step further and investigated the function of lung IM during latent TB infection (LTBI) utilizing a previously established mouse model of paucibacillary Mtb infection. In combination with the clodronate liposome depletion approach, they found that clodronate liposome-mediated transient IM depletion led to an increased relapse ratio of TB compared to mice that received the control liposome. Further analysis on cellular composition and cytokine levels in the lungs from different groups of mice revealed elevated inflammatory cytokines in the relapsed mice. Overall, this study uncovered the role of IM in controlling LTBI and preventing relapse, which is significant as the mechanism of TB relapse is largely unclear. The work, however, is descriptive and mostly correlated. The study is well-designed and the conclusions are supported by the data.

Major comments:
1. The study is largely based on the utilization of clodronate liposome to deplete monocytes and monocyte-derived cells, mainly IMs and monocyte-derived dendritic cells. It is known that this approach is relatively crude and has specificity issues. In certain contexts, it can even increase inflammation in the tissue. Such effects need to be acknowledged and elaborated in the discussion.

2. In Figure 2 A and B, Figure 3 B and D, please also include the absolute cell numbers which should reflect more directly in term of cellular alterations under different conditions.

3. In Figure 2J, the AM population in the uninfected t-SNE plot is very small. Please double check this as AMs is the major population of immune cells in uninfected mice. Additionally, there is a large cell population in the light blue color without a legend. What are those cells? Please define them. Lastly, eosinophils are missing in the current analysis. They can be defined by SiglecF+ CD11b+, which have already been included in the current flow cytometry analysis. They have been linked to Mtb control recently. It would be important to know their status in this study.

4. The increased number of neutrophils post-depletion correlated with the elevated KC and MIP-1a. Both of them are responsible for the recruitment of neutrophils. So it is possible that the transient increase in neutrophils promotes or at least contributes to the relapse. In particular, neutrophils are permissive cells and can act as cellular niches during Mtb infection. A further discussion is worth including in the discussion section.

5. In Figure 3E, how about the levels of IFNg and TNFa at the time of relapse? Please include these data if available. Any alterations of these two cytokines, especially IFNg may reflect the impact on T cell responses.

Minor comments:
1. Please include a detailed description of the mouse model of paucibacillary Mtb infection. This is missing in the method section.

2. In the legend of Figure 1, liposomes were injected every two or three days, which is inconsistent with the description in the Method section (every 3-4 days). Please correct this information.

3. Measuring various cytokines in the lung does not provide any direct assessment on the activity of macrophages and neutrophils. Please remove the sentence.

4. In Supp Figure 2, please include legends of the name for each cell population so it would be easier to interpret the changes of each marker.

Referee #2 (Remarks for Author):

This manuscript reports results of studies to determine the impact of intravenous clodronate administration to mice in an established model of latent infection with M. tuberculosis. The report focuses on 'interstitial macrophages' and observes that clodronate administration during latent M. tuberculosis infection is associated with increased regrowth of M. tuberculosis. The authors conclude that interstitial macrophages (defined here as CD45+Ly6G-CD11b+Siglec-F-CX3CR1+ lung cells) contribute to control of M. tuberculosis during latent infection. While the major question addressed is significant and the observations are original, the potential value of the findings is limited by multiple technical shortcomings as detailed below.
• What are the major claims and how significant are they?
The major claim is that 'interstitial macrophages' (defined by a low-resolution flow cytometry approach) provide protection from progressive M. tuberculosis infection in a model of TB latency. The significance of this major claim is low, for several reasons, most of which are technical. One major technical shortcoming is the low-resolution approach to defining lung leukocyte subsets, including 'interstitial macrophages' that are the focus of attention in the manuscript. There are multiple published higher resolution approaches with additional flow cytometry markers (PMIDs 28257233, 31481690, 32220976, 30872492) including in M. tuberculosis infection (PMIDs 38977711, 32544188, 38701094, 30365557), so it is difficult to place the findings in this work in the context of prior related work.
• Are the claims novel and convincing?
The claims are novel, in that the host mononuclear cells that contribute to maintenance of M. tuberculosis latency have not been studied previously. The claims regarding the impact of depleting interstitial macrophages throughout the manuscript are made less convincing by the finding that intravenouse clodronate liposome administration also depleted the cell population that the authors term 'recruited macrophages' (Figure 2). Despite nearly equivalent depletion of 'interstitial macrophages' and 'recruited macrophages', the manuscript only features interstitial macrophages in the remainder of the text, figures, and manuscript title. Addtional shortcomings that make the results less convincing include the failure to distinguish intravascular mononuclear cells from those in the lung parenchyma by administering labeled anti-CD45 antibody intravenously prior to euthanasia, and by the use of cell frequencies rather than cell numbers in presenting the data. The latter is of particular importance when the experiments involve depletion of a given cell subset in the population. A further shortcoming is the lack of efforts to address whether the population of macrophages of interest harbor M. tuberculosis during latency (admittedly difficult) or during relapse of infection.
• Are the claims appropriately discussed in the context of earlier literature?
No; see above

• Is the study important to the field?
Not especially, considering the technical flaws
• Does the paper stand out in some way from the others in its field?
No
• Is it also of interest to more than a specialized audience?
Not likely
• Are there other experiments that would strengthen the paper?
Resolution of the technical flaws noted above.

As a service to authors, EMBO provides authors with the possibility to transfer a manuscript that one journal cannot offer to publish to another EMBO publication. The full manuscript and if applicable, reviewers reports are automatically sent to the receiving journal to allow for fast handling and a prompt decision on your manuscript. For more details of this service, and to transfer your manuscript to another EMBO title please click on Link Not Available

Please do not share this URL as it will give anyone who clicks it access to your account.

***** Reviewer's comments *****

Referee #1 (Comments on Novelty/Model System for Author):

The technical quality of this manuscript is high as it includes necessary controls and appropriate statistical methods for data analysis. While the novelty of the study is medium as it is descriptive without a deep mechanistic analysis, it uncovered a previously undefined role of lung interstitial macrophages in controlling TB relapse during latent TB.

Referee #1 (Remarks for Author):

It has been known that different lung macrophage populations have distinct functions at the early stage of Mtb infection. Lung alveolar macrophages (AM) function as a permissive niche facilitating the growth and dissemination of Mtb whereas interstitial macrophages (IM) are more restrictive for Mtb during the first two weeks of infection. This study took one step further and investigated the function of lung IM during latent TB infection (LTBI) utilizing a previously established mouse model of paucibacillary Mtb infection. In combination with the clodronate liposome depletion approach, they found that clodronate liposome-mediated transient IM depletion led to an increased relapse ratio of TB compared to mice that received the control liposome. Further analysis on cellular composition and cytokine levels in the lungs from different groups of mice revealed elevated inflammatory cytokines in the relapsed mice. Overall, this study uncovered the role of IM in controlling LTBI and preventing relapse, which is significant as the mechanism of TB relapse is largely unclear. The work, however, is descriptive and mostly correlated. The study is well-designed and the conclusions are supported by the data.

This study is descriptive, but as this reviewer points out, it uncovered a novel role of pulmonary recruited and interstitial macrophages in controlling TB relapse. Future studies will be aimed at understanding the underlying mechanisms.

Major comments:

1. The study is largely based on the utilization of clodronate liposome to deplete monocytes and monocyte-derived cells, mainly IMs and monocyte-derived dendritic cells. It is known that this approach is relatively crude and has specificity issues. In certain contexts, it can even increase inflammation in the tissue. Such effects need to be acknowledged and elaborated in the discussion.

We are aware that the approach is relatively crude and carefully measured the impact on other immune cells besides IMs. Importantly for our study, alveolar macrophages and neutrophils were not depleted. We also included control liposomes to account for possible inflammation. We will acknowledge and discussed the limitations of this approach.

2. In Figure 2 A and B, Figure 3 B and D, please also include the absolute cell numbers which should reflect more directly in term of cellular alterations under different conditions.

We did not set up the experiment to recover all cells from the lungs. It was critical to utilize part of the lung tissue to assess bacterial burden, and we typically acquire more cells on the flow cytometer from the clodronate-liposome treated groups that the control groups. Therefore, the absolute counts are not reflective of cellular alterations and need to be corrected by the frequency of live cells for each population.

3. In Figure 2J, the AM population in the uninfected t-SNE plot is very small. Please double check this as AMs is the major population of immune cells in uninfected mice. Additionally, there is a large cell population in the light blue color without a legend. What are those cells? Please define them. Lastly, eosinophils are missing in the current analysis. They can be defined by SiglecF+ CD11b+, which have already been included in the current flow cytometry analysis. They have been linked to Mtb control recently. It would be important to know their status in this study.

We consistently recover 1-5% AMs from the population of CD45+ cells. With our tissue digestion protocol, we may be losing some AMs that would be recovered by bronchoalveolar lavage. Of note, the % of AMs we report is the % of all CD45+ cells, not of the whole myeloid compartment.

The light blue color in the t-SNE plots (cluster 11) represents an *undefined* population according to the markers, but they are likely naïve or resting T cells considering that they are negative for myeloid cell markers (e.g. macrophages, neutrophils, dendritic cells). We have added this to the results section (panel is now **Figure** 2K).

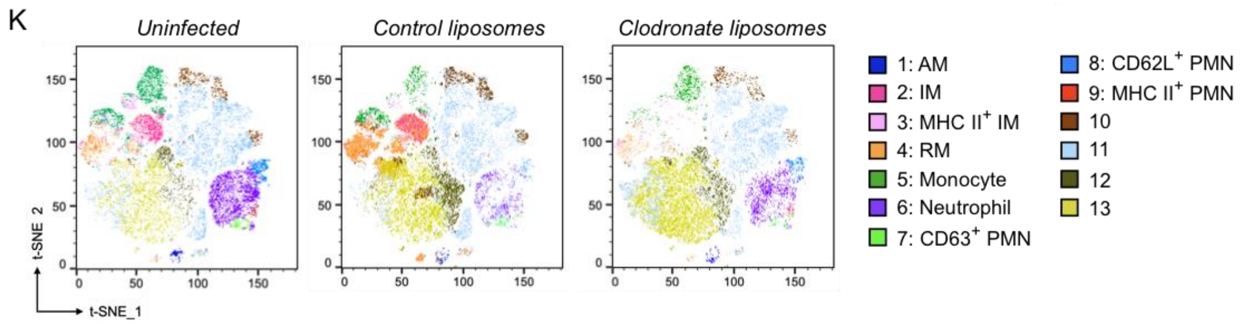

We generated the data and observed no significant difference in the frequency of eosinophils in mice treated with control compared to clodronate liposomes.

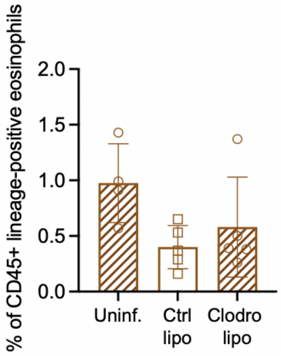

4. The increased number of neutrophils post-depletion correlated with the elevated KC and MIP-1a. Both of them are responsible for the recruitment of neutrophils. So it is possible that the transient increase in neutrophils promotes or at least contributes to the relapse. In particular, neutrophils are permissive cells and can act as cellular niches during Mtb infection. A further discussion is worth including in the discussion section.

This is a great point. We discussed the possibility that neutrophils contributed to relapse.

5. In Figure 3E, how about the levels of IFNg and TNFa at the time of relapse? Please include these data if available. Any alterations of these two cytokines, especially IFNg may reflect the impact on T cell responses.

We measured IFN-γ and TNF in the lung and this time point. Below are the data for all cytokines tested. There were no statistically significant changes in the levels of IFN-γ and TNF in mice that relapsed. This has been added to Supplemental Figure 3B.

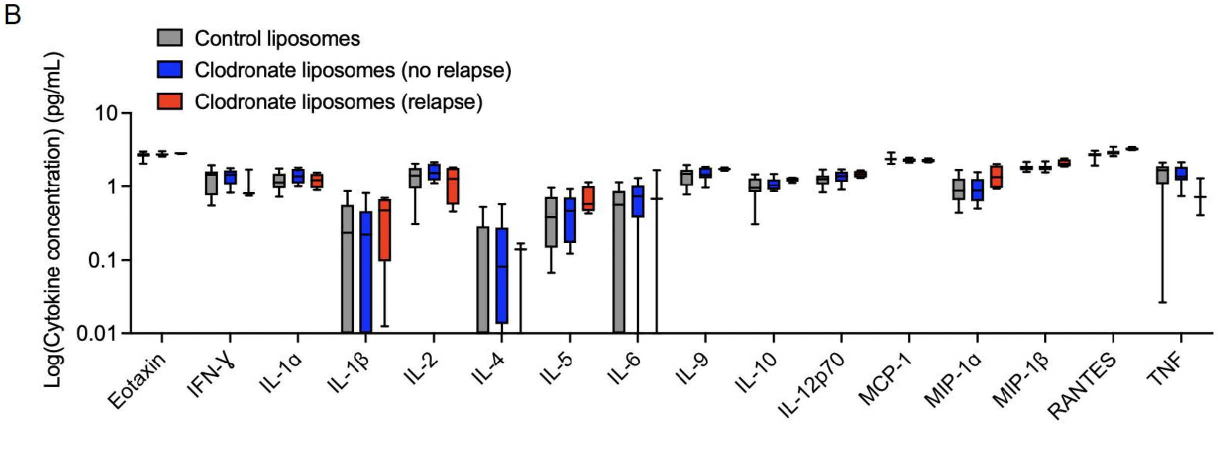

Minor comments:

1. Please include a detailed description of the mouse model of paucibacillary Mtb infection. This is missing in the method section.

We added this in the Materials and Methods.

2. In the legend of Figure 1, liposomes were injected every two or three days, which is inconsistent with the description in the Method section (every 3-4 days). Please correct this information.

Thank you for catching this inconsistency. Mice were injected every two to three days. We corrected this in the manuscript.

3. Measuring various cytokines in the lung does not provide any direct assessment on the activity of macrophages and neutrophils. Please remove the sentence.

We removed the sentence.

4. In Supp Figure 2, please include legends of the name for each cell population so it would be easier to interpret the changes of each marker.

This has instead been incorporated into Figure 2. Along with panel K, we believe that the information requested is best suited in Figure 2L, as shown below.

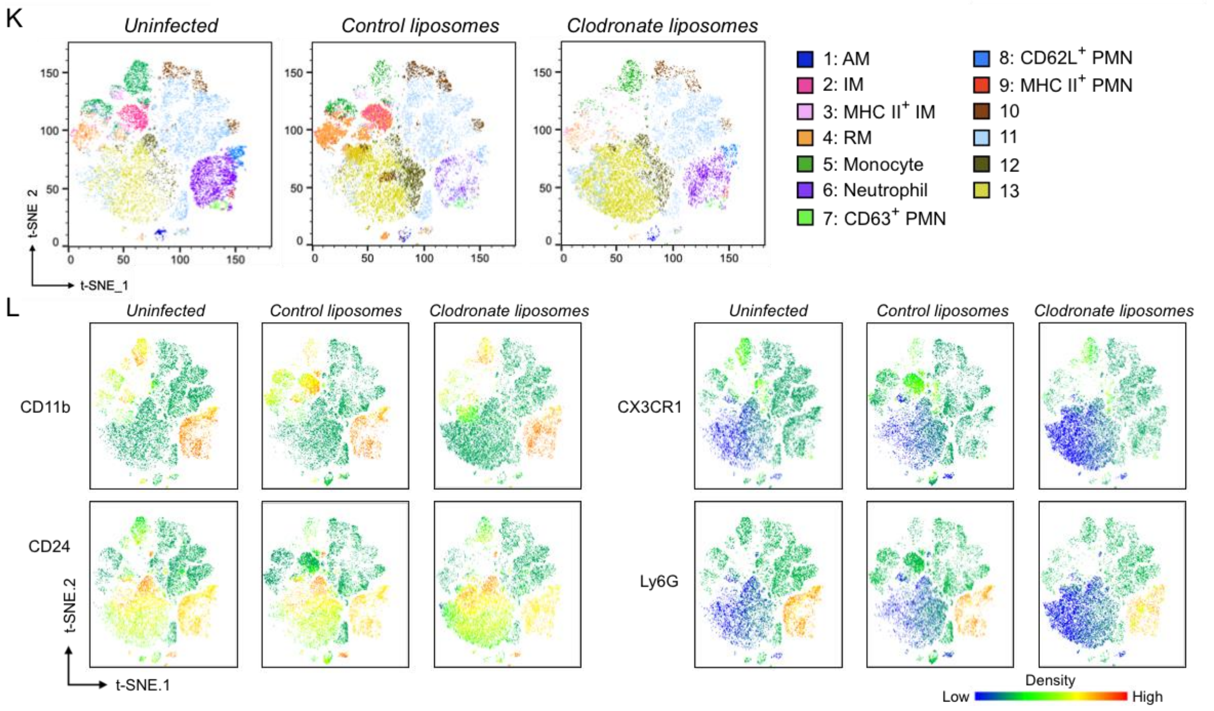

Referee #2 (Remarks for Author):

This manuscript reports results of studies to determine the impact of intravenous clodronate administration to mice in an established model of latent infection with M. tuberculosis. The report focuses on 'interstitial macrophages' and observes that clodronate administration during latent M. tuberculosis infection is associated with increased regrowth of M. tuberculosis. The authors conclude that interstitial macrophages (defined here as CD45+Ly6G-CD11b+Siglec-F-CX3CR1+ lung cells) contribute to control of M. tuberculosis during latent infection. While the major question addressed is significant and the observations are original, the potential value of the findings is limited by multiple technical shortcomings as detailed below.

We appreciate that the reviewer acknowledges that our work addresses a significant question and based on the comments below, it appears that there are two technical shortcomings, one of which we believe is not so much a technical issue rather than the choice of flow cytometry markers to define macrophage populations.

• *What are the major claims and how significant are they?*

The major claim is that 'interstitial macrophages' (defined by a low-resolution flow cytometry approach) provide protection from progressive M. tuberculosis infection in a model of TB latency. The significance of this major claim is low, for several reasons, most of which are technical. One major technical shortcoming is the low-resolution approach to defining lung leukocyte subsets, including 'interstitial macrophages' that are the focus of attention in the manuscript. There are multiple published higher resolution approaches with additional flow cytometry markers (PMIDs 28257233, 31481690, 32220976, 30872492) including in M. tuberculosis infection (PMIDs 38977711, 32544188, 38701094, 30365557), so it is difficult to place the findings in this work in the context of prior related work.

Prior related work by Dr. David Russell's group (Huang et al., 2018 *JEM*) revealed the importance of interstitial macrophages in controlling Mtb in an acute mouse model of tuberculosis infection. We built upon these findings by utilizing a paucibacillary mouse model of latent TB infection (Su et al., 2021 *JEM*) to further study the function of interstitial macrophages in restricting Mtb during latent infection. The markers we used to define recruited (which give rise to interstitial macrophages within the lung tissue) and interstitial macrophages are sufficient to distinguish them from alveolar macrophages and other immune cells. Additional markers such as CD64, F4/80, MerTK, are not essential to differentiate interstitial macrophages from alveolar macrophages which was the goal of our analysis. Similarly, dividing recruited and interstitial macrophages into subpopulations, while interesting, would not alter the conclusions of this study.

• *Are the claims novel and convincing?*

The claims are novel, in that the host mononuclear cells that contribute to maintenance

of M. tuberculosis latency have not been studied previously. The claims regarding the impact of depleting interstitial macrophages throughout the manuscript are made less convincing by the finding that intravenous clodronate liposome administration also depleted the cell population that the authors term 'recruited macrophages' (Figure 2). Despite nearly equivalent depletion of 'interstitial macrophages' and 'recruited macrophages', the manuscript only features interstitial macrophages in the remainder of the text, figures, and manuscript title.

We broadened the conclusion to highlight the importance of recruited and interstitial macrophages for control of TB latency.

Additional shortcomings that make the results less convincing include the failure to distinguish intravascular mononuclear cells from those in the lung parenchyma by administering labeled anti-CD45 antibody intravenously prior to euthanasia, and by the use of cell frequencies rather than cell numbers in presenting the data. The latter is of particular importance when the experiments involve depletion of a given cell subset in the population. A further shortcoming is the lack of efforts to address whether the population of macrophages of interest harbor M. tuberculosis during latency (admittedly difficult) or during relapse of infection.

This is a good suggestion. Unfortunately, we did not include this analysis. We will add it in future work. We did not recover all lung cells for flow cytometry, because it was critical to measure bacterial burden to assess relapse. Although we have tried very hard, we have not been able to detect Mtb during latent infection. It will be informative to determine which macrophage population(s) harbor Mtb during relapse, but this is also challenging to address given that only a small number of mice relapse and it is unknown which animal has relapse TB when they are sacrificed. We hope to use a fluorescent Mtb strain to identify infected cells in the future, but this requires new strain construction and a new mouse infection experiment.

25th Feb 2026

Dear Prof. Ehrt,

Thank you for the submission of your revised manuscript to EMBO Molecular Medicine. We have now heard back from two referee who we asked to re-evaluate your manuscript. As you will see from the reports pasted below, referee #1 supports publication of the manuscript after minor revision, while referee #2 remains critical regarding the technical issues. After an editorial discussion we agreed that all referee's concerns are appropriately addressed and that referee #1 minor point should be implemented in the next revised version. Therefore, I am pleased to inform you that we will be able to accept your manuscript pending the following final amendments:

- Please implement referee #1 suggestion to more accurately reflect the presented data.
- Please note that your manuscript will be published as a Report article. Please check our Author Guidelines for more information https://link.springer.com/journal/44321/submission-guidelines#cms-Article-types
- Please reformat your manuscript in accordance with the journal's requirements. Please check the points below as well as our Author Guidelines for more information https://www.embopress.org/page/journal/17574684/authorguide#manuscriptpreparation

I look forward to receiving a revised version of your manuscript as soon as possible.

Yours sincerely,

Zeljko Durdevic

Zeljko Durdevic
Senior Editor
EMBO Molecular Medicine

*** Instructions to submit your revised manuscript ***

*** PLEASE NOTE *** As part of the EMBO Publications transparent editorial process initiative (see our Editorial at https://www.embopress.org/doi/pdf/10.1002/emmm.201000094), EMBO Molecular Medicine will publish online a Review Process File to accompany accepted manuscripts.

In the event of acceptance, this file will be published in conjunction with your paper and will include the anonymous referee reports, your point-by-point response and all pertinent correspondence relating to the manuscript. If you do NOT want this file to be published, please inform the editorial office at contact@embomolmed.org.

*****

When preparing your revised manuscript, please refer to our guidelines: https://link.springer.com/journal/44321/submission-guidelines#cms-Revised-submissions. We perform an initial quality control of all revised manuscripts before re-review; failure to include requested items will delay the evaluation of your revision.

We require:

1) A .docx formatted version of the manuscript text (including legends for main figures, EV figures and tables). Please make sure that the changes are highlighted to be clearly visible.

2) Individual production quality figure files as .eps, .tif, .jpg (one file per figure). For guidance, download the 'Figure Guide PDF': https://media.springernature.com/original/springer-cms/rest/v1/content/27825798/data/v1.

3) A .docx formatted letter INCLUDING the reviewers' reports and your detailed point-by-point responses to their comments. As

part of the EMBO Press transparent editorial process, the point-by-point response is part of the Review Process File (RPF), which will be published alongside your paper.

4) A complete author checklist, which you can download from our author guidelines. Please insert information in the checklist that is also reflected in the manuscript. The completed author checklist will also be part of the RPF.

5) Please note that all corresponding authors are required to supply an ORCID ID for their name upon submission of a revised manuscript.

6) It is mandatory to include a 'Data Availability' section after the Materials and Methods. Before submitting your revision, primary datasets produced in this study need to be deposited in an appropriate public database, and the accession numbers and database listed under 'Data Availability'. Please remember to provide a reviewer password if the datasets are not yet public.

In case you have no data that requires deposition in a public database, please state so in this section. Note that the Data Availability Section is restricted to new primary data that are part of this study.

7) For data quantification: please specify the name of the statistical test used to generate error bars and P values, the number (n) of independent experiments (specify technical or biological replicates) underlying each data point and the test used to calculate p-values in each figure legend. The figure legends should contain a basic description of n, P and the test applied. Graphs must include a description of the bars and the error bars (s.d., s.e.m.).

8) At EMBO Press we ask authors to provide source data for the main manuscript figures. You will receive a separate email with instructions for providing source data with your revised manuscript, including how to upload and organize the files.

9) Our journal encourages inclusion of *data citations in the reference list* to directly cite datasets that were re-used and obtained from public databases. Data citations in the article text are distinct from normal bibliographical citations and should directly link to the database records from which the data can be accessed. In the main text, data citations are formatted as follows: "Data ref: Smith et al, 2001" or "Data ref: NCBI Sequence Read Archive PRJNA342805, 2017". In the Reference list, data citations must be labeled with "[DATASET]". A data reference must provide the database name, accession number/identifiers and a resolvable link to the landing page from which the data can be accessed at the end of the reference.

10) We replaced Supplementary Information with Expanded View (EV) Figures and Tables that are collapsible/expandable online. A maximum of 5 EV Figures can be typeset. EV Figures should be cited as 'Figure EV1, Figure EV2" etc... in the text and their respective legends should be included in the main text after the legends of regular figures.

- For the figures that you do NOT wish to display as Expanded View figures, they should be bundled together with their legends in a single PDF file called *Appendix*, which should start with a short Table of Content. Appendix figures should be referred to in the main text as: "Appendix Figure S1, Appendix Figure S2" etc.

- Additional Tables/Datasets should be labeled and referred to as Table EV1, Dataset EV1, etc. Legends have to be provided in a separate tab in case of .xls files. Alternatively, the legend can be supplied as a separate text file (README) and zipped together with the Table/Dataset file.

11) The paper explained: EMBO Molecular Medicine articles are accompanied by a summary of the articles to emphasize the major findings in the paper and their medical implications for the non-specialist reader. Please provide a draft summary of your article highlighting

- the medical issue you are addressing,

- the results obtained and

- their clinical impact.

This may be edited to ensure that readers understand the significance and context of the research. Please refer to any of our published articles for an example.

12) Author contributions: You will be asked to provide CRediT (Contributor Role Taxonomy) terms in the submission system. These replace a narrative author contribution section in the manuscript.

13) A Conflict of Interest statement should be provided in the main text.

14) Every published paper includes a 'Synopsis' to further enhance discoverability. Synopses are displayed on the journal webpage and are freely accessible to all readers. They include a short stand first (maximum of 300 characters, including space)

as well as 2-5 one-sentences bullet points that summarizes the paper. Please write the bullet points to summarize the key NEW findings. They should be designed to be complementary to the abstract - i.e. not repeat the same text. We encourage inclusion of key acronyms and quantitative information (maximum of 30 words / bullet point). Please use the passive voice. Please attach these in a separate file or send them by email, we will incorporate them accordingly.

Please also suggest a striking image or visual abstract to illustrate your article as a PNG file 550 px wide x 300-600 px high.

15) Include a Reagents and Tools Table as part of the Methods section, which can be downloaded from our author guidelines.

EMBO Molecular Medicine has a "scooping protection" policy, whereby similar findings that are published by others during review or revision are not a criterion for rejection. Should you decide to submit a revised version, I do ask that you get in touch after three months if you have not completed it, to update us on the status.

*Additional important information regarding Figures

Each figure should be given in a separate file and should have the following resolution:
Graphs 800-1,200 DPI
Photos 400-800 DPI
Colour (only CMYK) 300-400 DPI"

Figures are not edited by the production team. All lettering should be the same size and style; figure panels should be indicated by capital letters (A, B, C etc). Gridlines are not allowed except for log plots. Figures should be numbered in the order of their appearance in the text with Arabic numerals. Each Figure must have a separate legend and a caption is needed for each panel.

*Additional important information regarding figures and illustrations can be found at
https://media.springernature.com/original/springer-cms/rest/v1/content/27825798/data/v1

***** Reviewer's comments *****

Referee #1 (Comments on Novelty/Model System for Author):

This manuscript investigate the function of lung interstitial macrophages (IMs) during latent TB infection utilizing an established TB relapse model. The technical quality of this manuscript is high as the relapse TB model it used has been published previously and represents key features of TB relapse. While IMs has been recognized for their more restrictive nature of controlling Mtb replication at the early stage of infection, their roles in regulating TB relapse is unclear. Lastly, this report revealed a novel mechanism that contributes to TB relapse independent of impaired T cell responses, implicating the key role of altered myeloid cell dynamics in the disease outcome.

Referee #1 (Remarks for Author):

This study examined the role of lung interstitial macrophages during latent TB infection using a previously established mouse model that recapitulates TB relapse. While clodronate liposome-mediated depletion of interstitial macrophages has been considered a relatively crude method, it is essential to recognize that the depletion of lung interstitial macrophages is technically challenging. Importantly, this study has carefully ruled out the possibility of impaired functions of neutrophils, a newly observed off-target effect of clodronate liposome. The authors have included some new analysis on the frequency of eosinophils. Overall, all my previous points have been addressed. One minor point that I feel the authors need to acknowledge is the issue with distinguishing interstitial macrophages from recruited macrophages. Both of these populations are reduced upon clodronate liposome administration. Therefore, both of these two monocyte-derived macrophages may contribute to prevent TB relapse in this model. So revising the title and the tone throughout the manuscript may reflect the data more accurately.

Referee #2 (Remarks for Author):

The authors have responded to major points in the original review in a rebuttal but have not resolved the concerns regarding the technical issues.

The authors addressed the editorial issues.

26th Mar 2026

Dear Prof. Ehrt,

Thank you for the submission of your revised manuscript to EMBO Molecular Medicine. I am pleased to inform you that we will be able to accept your manuscript pending the following final amendments:

1) Authors: Please change the author names to the full First name Last name in the manuscript text.
2) In the main manuscript file, please do the following:
- Please address all comments suggested by our data editors listed below:
o Figure legends:
1. Please define the annotated p values ****/***/**/* as well as provide the exact p-values for the same in the legend of figure 4C as appropriate.
2. Please note that the exact p values are not provided in the legends of figures 1C-F; 3D, E; EV2 A-C.
3. Please indicate the statistical test used for data analysis in the legends of figures 1F, 2J, 4C.
4. Please note that the box plots need to be defined in terms of minima, maxima, centre, bounds of box and whiskers, and percentile in the legends of figures 3E, EV3 B.
5. Please note that information related to n is missing in the legend of figure 4C.
6. Please note that the error bars are not defined in the legend of figure 4C.
- Please correct the order and the headings of the sections in the manuscript text to: Abstract / The Paper Explained / Introduction / Results and Discussion / Methods / Data Availability / Acknowledgements / Disclosure and Competing Interests Statement / References / Figure Legends / Tables / Expanded View Figure Legends
- Remove information about BioRender from the synopsis/synopsis image and add a dedicated "Graphics" section in the Methods, following this format:
Graphics:
(some of the... OR Figure #... OR synopsis) Graphics were created with BioRender.com.
- Indicate in legends exact n and exact p values, not a range, along with the statistical test used. To keep the figures "clear" some authors found providing an Appendix table Sx with all exact p-values preferable. You are welcome to do this if you want to.
- Author contributions: Please remove it from the manuscript and specify author contributions in our submission system. CRediT has replaced the traditional author contributions section because it offers a systematic machine-readable author contributions format that allows for more effective research assessment. You are encouraged to use the free text boxes beneath each contributing author's name to add specific details on the author's contribution. More information is available in our guide to authors:
https://www.embopress.org/page/journal/17574684/authorguide#authorshipguidelines
- Please correct the reference citation in the reference list. Where there are more than 10 authors on a paper, 10 will be listed, followed by "et al.". Also, please remove DOIs for published articles. Please check "Author Guidelines" for more information.
https://www.embopress.org/page/journal/17574684/authorguide#referencesformat
3) Source data: Please upload a completed source data checklist.
4) The Paper Explained: Please add it to the main manuscript file.
5) Synopsis:
- Synopsis image: Please upload it as a separate, high-resolution .jpeg file 550 px-wide x 300-600 pixels high to illustrate your article.
- Please check your synopsis text and image before submission with your revised manuscript. Please be aware that in the proof stage minor corrections only are allowed (e.g., typos).
6) As part of the EMBO Publications transparent editorial process (see our Editorial at http://embomolmed.embopress.org/content/2/9/329), EMBO Molecular Medicine will publish online a Review Process File (RPF) to accompany accepted manuscripts. This file will be published in conjunction with your paper and will include the anonymous referee reports, your point-by-point response and all pertinent correspondence relating to the manuscript. Let us know if you want to remove or not any figures from it prior to publication. Please note that the Authors checklist will be published at the end of the RPF.
7) Please provide a point-by-point letter INCLUDING my comments as well as the reviewer's reports and your detailed responses (as Word file).

I look forward to reading a new revised version of your manuscript as soon as possible.

Yours sincerely,

Zeljko Durdevic

Zeljko Durdevic

Senior Editor
EMBO Molecular Medicine

*** Instructions to submit your revised manuscript ***

*** PLEASE NOTE *** As part of the EMBO Publications transparent editorial process initiative (see our Editorial at https://www.embopress.org/doi/pdf/10.1002/emmm.201000094), EMBO Molecular Medicine will publish online a Review Process File to accompany accepted manuscripts.

In the event of acceptance, this file will be published in conjunction with your paper and will include the anonymous referee reports, your point-by-point response and all pertinent correspondence relating to the manuscript. If you do NOT want this file to be published, please inform the editorial office at contact@embomolmed.org.

*****

When preparing your revised manuscript, please refer to our guidelines: https://link.springer.com/journal/44321/submission-guidelines#cms-Revised-submissions. We perform an initial quality control of all revised manuscripts before re-review; failure to include requested items will delay the evaluation of your revision.

We require:

1) A .docx formatted version of the manuscript text (including legends for main figures, EV figures and tables). Please make sure that the changes are highlighted to be clearly visible.

2) Individual production quality figure files as .eps, .tif, .jpg (one file per figure). For guidance, download the 'Figure Guide PDF': https://media.springernature.com/original/springer-cms/rest/v1/content/27825798/data/v1.

3) A .docx formatted letter INCLUDING the reviewers' reports and your detailed point-by-point responses to their comments. As part of the EMBO Press transparent editorial process, the point-by-point response is part of the Review Process File (RPF), which will be published alongside your paper.

4) A complete author checklist, which you can download from our author guidelines. Please insert information in the checklist that is also reflected in the manuscript. The completed author checklist will also be part of the RPF.

5) Please note that all corresponding authors are required to supply an ORCID ID for their name upon submission of a revised manuscript.

6) It is mandatory to include a 'Data Availability' section after the Materials and Methods. Before submitting your revision, primary datasets produced in this study need to be deposited in an appropriate public database, and the accession numbers and database listed under 'Data Availability'. Please remember to provide a reviewer password if the datasets are not yet public.

In case you have no data that requires deposition in a public database, please state so in this section. Note that the Data Availability Section is restricted to new primary data that are part of this study.

7) For data quantification: please specify the name of the statistical test used to generate error bars and P values, the number (n) of independent experiments (specify technical or biological replicates) underlying each data point and the test used to calculate p-values in each figure legend. The figure legends should contain a basic description of n, P and the test applied. Graphs must include a description of the bars and the error bars (s.d., s.e.m.).

8) At EMBO Press we ask authors to provide source data for the main manuscript figures. You will receive a separate email with instructions for providing source data with your revised manuscript, including how to upload and organize the files.

9) Our journal encourages inclusion of *data citations in the reference list* to directly cite datasets that were re-used and obtained from public databases. Data citations in the article text are distinct from normal bibliographical citations and should directly link to the database records from which the data can be accessed. In the main text, data citations are formatted as follows: "Data ref: Smith et al, 2001" or "Data ref: NCBI Sequence Read Archive PRJNA342805, 2017". In the Reference list,

data citations must be labeled with "[DATASET]". A data reference must provide the database name, accession number/identifiers and a resolvable link to the landing page from which the data can be accessed at the end of the reference.

10) We replaced Supplementary Information with Expanded View (EV) Figures and Tables that are collapsible/expandable online. A maximum of 5 EV Figures can be typeset. EV Figures should be cited as 'Figure EV1, Figure EV2" etc... in the text and their respective legends should be included in the main text after the legends of regular figures.

- For the figures that you do NOT wish to display as Expanded View figures, they should be bundled together with their legends in a single PDF file called *Appendix*, which should start with a short Table of Content. Appendix figures should be referred to in the main text as: "Appendix Figure S1, Appendix Figure S2" etc.

- Additional Tables/Datasets should be labeled and referred to as Table EV1, Dataset EV1, etc. Legends have to be provided in a separate tab in case of .xls files. Alternatively, the legend can be supplied as a separate text file (README) and zipped together with the Table/Dataset file.

11) The paper explained: EMBO Molecular Medicine articles are accompanied by a summary of the articles to emphasize the major findings in the paper and their medical implications for the non-specialist reader. Please provide a draft summary of your article highlighting

- the medical issue you are addressing,

- the results obtained and

- their clinical impact.

This may be edited to ensure that readers understand the significance and context of the research. Please refer to any of our published articles for an example.

12) Author contributions: You will be asked to provide CRediT (Contributor Role Taxonomy) terms in the submission system. These replace a narrative author contribution section in the manuscript.

13) A Conflict of Interest statement should be provided in the main text.

14) Every published paper includes a 'Synopsis' to further enhance discoverability. Synopses are displayed on the journal webpage and are freely accessible to all readers. They include a short stand first (maximum of 300 characters, including space) as well as 2-5 one-sentences bullet points that summarizes the paper. Please write the bullet points to summarize the key NEW findings. They should be designed to be complementary to the abstract - i.e. not repeat the same text. We encourage inclusion of key acronyms and quantitative information (maximum of 30 words / bullet point). Please use the passive voice. Please attach these in a separate file or send them by email, we will incorporate them accordingly.

Please also suggest a striking image or visual abstract to illustrate your article as a PNG file 550 px wide x 300-600 px high.

15) Include a Reagents and Tools Table as part of the Methods section, which can be downloaded from our author guidelines.

EMBO Molecular Medicine has a "scooping protection" policy, whereby similar findings that are published by others during review or revision are not a criterion for rejection. Should you decide to submit a revised version, I do ask that you get in touch after three months if you have not completed it, to update us on the status.

*Additional important information regarding Figures

Each figure should be given in a separate file and should have the following resolution:
Graphs 800-1,200 DPI
Photos 400-800 DPI
Colour (only CMYK) 300-400 DPI"

Figures are not edited by the production team. All lettering should be the same size and style; figure panels should be indicated by capital letters (A, B, C etc). Gridlines are not allowed except for log plots. Figures should be numbered in the order of their appearance in the text with Arabic numerals. Each Figure must have a separate legend and a caption is needed for each panel.

*Additional important information regarding figures and illustrations can be found at
https://media.springernature.com/original/springer-cms/rest/v1/content/27825798/data/v1

The authors addressed the remaining formatting issues.

13th Apr 2026

Dear Prof. Ehrt,

We are pleased to inform you that your manuscript is accepted for publication and is now being sent to our publisher to be included in the next available issue of EMBO Molecular Medicine.

Your manuscript will be processed for publication by EMBO Press. It will be copy edited and you will receive page proofs prior to publication. Please note that you will be contacted by Springer Nature Author Services to complete licensing and payment information.

You may qualify for financial assistance for your publication charges - either via a Springer Nature fully open access agreement or an EMBO initiative. Check your eligibility: https://link.springer.com/journal/44321/how-to-publish-with-us

Should you be planning a Press Release on your article, please get in contact with embo_production@springernature.com as early as possible in order to coordinate publication and release dates.

If you have any questions, please do not hesitate to contact the Editorial Office. Thank you for your contribution to EMBO Molecular Medicine.

Yours sincerely,
Zeljko Durdevic

Zeljko Durdevic
Senior Editor
EMBO Molecular Medicine

>>> Please note that it is EMBO Molecular Medicine policy for the transcript of the editorial process (containing referee reports and your response letter) to be published as an online supplement to each paper. If you do NOT want this, you will need to inform the Editorial Office via email immediately. More information is available here: https://link.springer.com/partners/embo-press/editorial-policies#Peer%20review

EMBO Press Author Checklist

Corresponding Author Name: Sabine Ehrt	
Journal Submitted to: EMBO Molecular Medicine	
Manuscript Number: EMM-2025-22646-V2	

USEFUL LINKS FOR COMPLETING THIS FORM
The EMBO Journal - Author Guidelines
EMBO Reports - Author Guidelines
Molecular Systems Biology - Author Guidelines
EMBO Molecular Medicine - Author Guidelines

Reporting Checklist for Life Science Articles (updated January
This checklist is adapted from Materials Design Analysis Reporting (MDAR) Checklist for Authors. MDAR establishes a minimum set of requirements in transparent reporting in the life sciences (see Statement of Task: 10.31222/osf.io/9sm4x). Please follow the journal's guidelines in preparing your
Please note that a copy of this checklist will be published alongside your article.

Abridged guidelines for figures
1. Data
The data shown in figures should satisfy the following conditions:
- the data were obtained and processed according to the field's best practice and are presented to reflect the results of the experiments in an accurate and unbiased manner.
- ideally, figure panels should include only measurements that are directly comparable to each other and obtained with the same assay.
- plots include clearly labeled error bars for independent experiments and sample sizes. Unless justified, error bars should not be shown for technical
- if n<5, the individual data points from each experiment should be plotted. Any statistical test employed should be justified.
- Source Data should be included to report the data underlying figures according to the guidelines set out in the authorship guidelines on Data

2. Captions
Each figure caption should contain the following information, for each panel where they are relevant:
- a specification of the experimental system investigated (eg cell line, species name).
- the assay(s) and method(s) used to carry out the reported observations and measurements.
- an explicit mention of the biological and chemical entity(ies) that are being measured.
- an explicit mention of the biological and chemical entity(ies) that are altered/varied/perturbed in a controlled manner.
- the exact sample size (n) for each experimental group/condition, given as a number, not a range;
- a description of the sample collection allowing the reader to understand whether the samples represent technical or biological replicates (including how many animals, litters, cultures, etc.).
- a statement of how many times the experiment shown was independently replicated in the laboratory.
- definitions of statistical methods and measures:
  - common tests, such as t-test (please specify whether paired vs. unpaired), simple $\chi 2$ tests, Wilcoxon and Mann-Whitney tests, can be unambiguously identified by name only, but more complex techniques should be described in the methods section;
  - are tests one-sided or two-sided?
  - are there adjustments for multiple comparisons?
  - exact statistical test results, e.g., P values = x but not P values < x;
  - definition of 'center values' as median or average;
  - definition of error bars as s.d. or s.e.m.

Please complete ALL of the questions below.
Select "Not Applicable" only when the requested information is not relevant for your study.

Materials

Newly Created Materials	Information included in the manuscript?	In which section is the information available? (Reagents and Tools Table, Materials and Methods, Figures, Data Availability Section)
New materials and reagents need to be available; do any restrictions apply?	Not Applicable	

Antibodies	Information included in the manuscript?	In which section is the information available? (Reagents and Tools Table, Materials and Methods, Figures, Data Availability Section)
For **antibodies** provide the following information:<br>- Commercial antibodies: RRID (if possible) or supplier name, catalogue number and or/clone number<br>- Non-commercial: RRID or citation	Yes	Reagents and Tools Table, Materials and Methods

DNA and RNA sequences	Information included in the manuscript?	In which section is the information available? (Reagents and Tools Table, Materials and Methods, Figures, Data Availability Section)
**Short novel DNA or RNA including primers, probes**: provide the sequences.	Not Applicable	

Cell materials	Information included in the manuscript?	In which section is the information available? (Reagents and Tools Table, Materials and Methods, Figures, Data Availability Section)
**Cell lines:** Provide species information, strain. Provide accession number in repository **OR** supplier name, catalog number, clone number, and/**OR** RRID.	Not Applicable	
**Primary cultures:** Provide species, strain, sex of origin, genetic modification status.	Not Applicable	
Report if the cell lines were recently **authenticated** (e.g., by STR profiling) and tested for mycoplasma contamination.	Not Applicable	

Experimental animals	Information included in the manuscript?	In which section is the information available? (Reagents and Tools Table, Materials and Methods, Figures, Data Availability Section)
**Laboratory animals or Model organisms:** Provide species, strain, sex, age, genetic modification status. Provide accession number in repository **OR** supplier name, catalog number, clone number, **OR** RRID.	Yes	Reagents and Tools Table, Materials and Methods
**Animal observed in or captured from the field:** Provide species, sex, and age where possible.	Not Applicable	
Please detail **housing and husbandry conditions**.	Yes	Materials and Methods

Plants and microbes	Information included in the manuscript?	In which section is the information available? (Reagents and Tools Table, Materials and Methods, Figures, Data Availability Section)
**Plants:** provide species and strain, ecotype and cultivar where relevant, unique accession number if available, and source (including location for collected wild specimens).	Not Applicable	
**Microbes:** provide species and strain, unique accession number if available, and source.	Yes	Material and Methods

Human research participants	Information included in the manuscript?	In which section is the information available? (Reagents and Tools Table, Materials and Methods, Figures, Data Availability Section)
If collected and within the bounds of privacy constraints report on age, sex and gender or ethnicity for all study participants.	Not Applicable	

Core facilities	Information included in the manuscript?	In which section is the information available? (Reagents and Tools Table, Materials and Methods, Figures, Data Availability Section)
If your work benefited from core facilities, was their service mentioned in the acknowledgments section?	Yes	Acknowledgements

Design

**Study protocol**	**Information included in the manuscript?**	**In which section is the information available?** (Reagents and Tools Table, Materials and Methods, Figures, Data Availability Section)
If study protocol has been **pre-registered, provide DOI in the manuscript**. For clinical trials, provide the trial registration number **OR** cite DOI.	Not Applicable	
Report the **clinical trial registration number** (at ClinicalTrials.gov or equivalent), where applicable.	Not Applicable	

**Laboratory protocol**	**Information included in the manuscript?**	**In which section is the information available?** (Reagents and Tools Table, Materials and Methods, Figures, Data Availability Section)
Provide DOI OR other citation details if **external detailed step-by-step protocols** are available.	Not Applicable	

**Experimental study design and statistics**	**Information included in the manuscript?**	**In which section is the information available?** (Reagents and Tools Table, Materials and Methods, Figures, Data Availability Section)
Include a statement about **sample size** estimate even if no statistical methods were used.	Yes	Table
Were any steps taken to minimize the effects of subjective bias when allocating animals/samples to treatment (e.g. **randomization procedure**)? If yes, have they been described?	Not Applicable	
Include a statement about **blinding** even if no blinding was done.	Yes	Materials and Methods (Statistical analyzes)
Describe **inclusion/exclusion criteria** if samples or animals were excluded from the analysis. Were the criteria pre-established? <br>If sample or data points were omitted from analysis, report if this was due to attrition or intentional exclusion and provide justification.	Yes	Materials and Methods (Statistical analyzes)
For every figure, are **statistical tests** justified as appropriate? Do the data meet the assumptions of the tests (e.g., normal distribution)? Describe any methods used to assess it. Is there an estimate of variation within each group of data? Is the variance similar between the groups that are being statistically compared?	Yes	Materials and Methods

**Sample definition and in-laboratory replication**	**Information included in the manuscript?**	**In which section is the information available?** (Reagents and Tools Table, Materials and Methods, Figures, Data Availability Section)
In the figure legends: state number of times the experiment was **replicated** in laboratory.	Yes	Figure legends
In the figure legends: define whether data describe **technical or biological replicates**.	Yes	Figure legends

Ethics

**Ethics**	**Information included in the manuscript?**	**In which section is the information available?** (Reagents and Tools Table, Materials and Methods, Figures, Data Availability Section)
Studies involving **human participants**: State details of **authority granting ethics approval** (IRB or equivalent committee(s), provide reference number for approval.	Not Applicable	
Studies involving **human participants**: Include a statement confirming that **informed consent** was obtained from all subjects and that the experiments conformed to the principles set out in the WMA Declaration of Helsinki and the Department of Health and Human Services Belmont Report.	Not Applicable	
Studies involving **human participants:** For publication of **patient photos**, include a statement confirming that consent to publish was obtained.	Not Applicable	
Studies involving experimental **animals**: State details of **authority granting ethics approval** (IRB or equivalent committee(s), provide reference number for approval. Include a statement of compliance with ethical regulations.	Yes	Materials and Methods
Studies involving **specimen and field samples:** State if relevant **permits** obtained, provide details of authority approving study; if none were required, explain why.	Not Applicable	

**Dual Use Research of Concern (DURC)**	**Information included in the manuscript?**	**In which section is the information available?** (Reagents and Tools Table, Materials and Methods, Figures, Data Availability Section)
Could your study fall under dual use research restrictions? Please check biosecurity documents and list of **select agents and toxins** (CDC): https://www.selectagents.gov/sat/list.htm	Not Applicable	
If you used a select agent, is the security level of the lab appropriate and reported in the manuscript?	Not Applicable	
If a study is subject to dual use research of concern regulations, is the name of the **authority granting approval and reference number** for the regulatory approval provided in the manuscript?	Not Applicable	

Reporting

The MDAR framework recommends adoption of discipline-specific guidelines, established and endorsed through community initiatives. Journals have their own policy about requiring specific guidelines and recommendations to complement MDAR.

**Adherence to community standards**	**Information included in the manuscript?**	**In which section is the information available?** (Reagents and Tools Table, Materials and Methods, Figures, Data Availability Section)
State if relevant guidelines or checklists (e.g., **ICMJE, MIBBI, ARRIVE, PRISMA**) have been followed or provided.	Not Applicable	
For **tumor marker prognostic studies,** we recommend that you follow the **REMARK** reporting guidelines (see link list at top right). See author guidelines, under 'Reporting Guidelines'. Please confirm you have followed these guidelines.	Not Applicable	
For **phase II and III randomized controlled trials**, please refer to the **CONSORT** flow diagram (see link list at top right) and submit the CONSORT checklist (see link list at top right) with your submission. See author guidelines, under 'Reporting Guidelines'. Please confirm you have submitted this list.	Not Applicable	

Data Availability

**Data availability**	**Information included in the manuscript?**	**In which section is the information available?** (Reagents and Tools Table, Materials and Methods, Figures, Data Availability Section)
Have **primary datasets** been deposited according to the journal's guidelines (see 'Data Deposition' section) and the respective accession numbers provided in the Data Availability Section?	Not Applicable	
Were **human clinical and genomic datasets** deposited in a public access-controlled repository in accordance to ethical obligations to the patients and to the applicable consent agreement?	Not Applicable	
Are **computational models** that are central and integral to a study available without restrictions in a machine-readable form? Were the relevant accession numbers or links provided?	Not Applicable	
If publicly available data were reused, provide the respective **data citations in the reference list**.	Not Applicable	
